# On the Origin of Enantioselectivity in Chiral Zeolite Asymmetric Catalyst GTM-3: Host–Guest Transfer of Chirality

**DOI:** 10.1021/acsami.4c14487

**Published:** 2024-09-25

**Authors:** Ramón de la Serna, Jaime Jurado-Sánchez, Jian Li, Carlos Márquez-Álvarez, Joaquín Pérez-Pariente, Luis Gómez-Hortigüela

**Affiliations:** † Instituto de Catálisis y Petroleoquímica, Consejo Superior de Investigaciones Científicas (ICP-CSIC), c/Marie Curie 2, Madrid 28049, Spain; ‡ State Key Laboratory of Coordination Chemistry, School of Chemistry and Chemical Engineering, Nanjing University, Nanjing 210023, China

**Keywords:** zeolite, chirality, enantioselective, asymmetric catalysis, absolute configuration

## Abstract

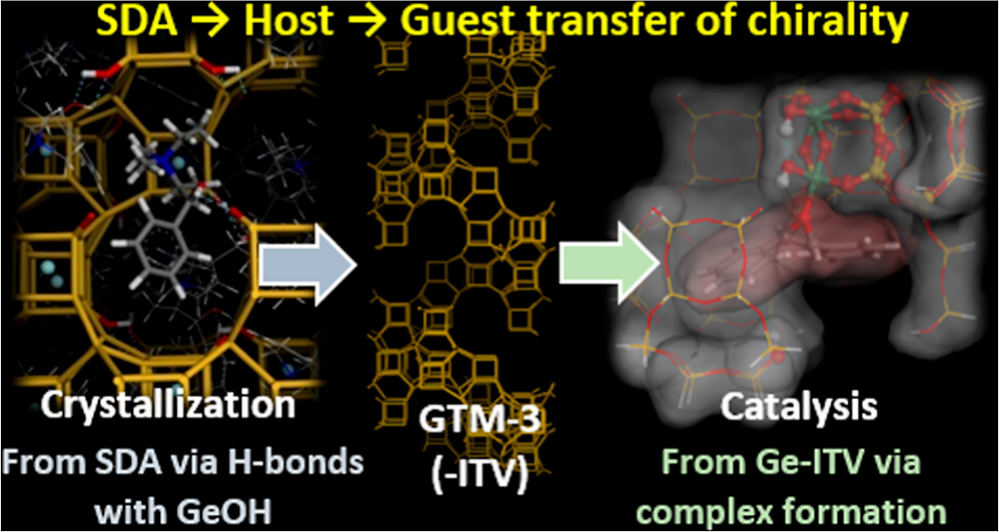

Knowledge of how extra-large-pore chiral zeolite asymmetric catalysts based on the -ITV framework imprint their chirality during a catalytic reaction is crucial in order to spread the scope for the catalytic enantioselective production of chiral compounds of interest. In this work, we have carried out a combined experimental and computational study on the catalytic activity of antipode GTM-3 catalysts during the ring-opening of *trans*-stilbene oxide with 1-butanol. Identification of the enantiomers of all the chiral species unraveled a surprising catalytic behavior: these chiral catalysts promote the transformation of one enantiomer of *trans*-stilbene oxide in the corresponding *unlike* product (with inversion of configuration of the attacked C) via an S_N_2 mechanism, and at the same time, the transformation of the other enantiomer of *trans*-stilbene oxide via an S_N_1-like mechanism into the *like* (with retention of configuration) and secondary products (diphenylacetaldehyde via *Meinwald* rearrangement and derived products). A computational study based on DFT + D methods suggested a potential explanation for this catalytic behavior, associated with a different orientation of *trans*-stilbene oxide enantiomers bound on the Ge(T7) positions in *d4r* units, which is stabilized by the development of intraframework H-bonds between the interrupted T7OH adjacent positions characteristic of this framework. Calculations suggest that each enantiomer of *trans*-stilbene oxide follows a different reaction pathway, one favoring the S_N_2 route by addition of butanol from the opposite side to form *unlike*-products, while the different orientation of the antipode enantiomer disfavors such S_N_2 route mainly by steric repulsions and at the same time favors the reaction toward the S_N_1 mechanism to give *like-* and secondary-products. Our study suggests that the strong enantioselectivity of GTM-3 catalysts for this reaction is associated with the particular orientation adopted by the chiral reactants within the chiral nanospace provided by the -ITV framework, similarly to what occurs with enzymes, and such preferential orientation is directly controlled by the asymmetric cavities where the reaction takes place, by the particular features of the Ge active sites in adjacent interrupted positions and by the presence of several framework OH groups in the nearby nanospace that interact with guest species. The experimental observations and the reaction mechanism proposed suggest that GTM-3 catalysts prepared from the (1*S*,2*S*) enantiomer of the *N*,*N*-ethyl-methyl-pseudoephedrinium organic agent should be enriched in the *P*4_3_32 enantiomorphic space group of the -ITV framework and GTM-3 prepared from the (1*R*,2*R*) enantiomer in the antipode *P*4_1_32. Interestingly, resolution of the absolute configuration of GTM-3 materials from 3D-electron diffraction data has been accomplished and confirms such an assignment, giving an average 82% enantio-enrichment in the corresponding chiral polymorph. Structure-solution of the location of the chiral structure-directing agents indicates that the transfer of chirality from the molecular component to the zeolite polymorph is governed by the development of strong H-bonds between the molecular hydroxyl group and the interrupted T(7)OH framework positions.

## 1 Introduction

Due to the crucial role of chirality in living organisms, the development of efficient technologies to enantioselectively prepare chiral compounds in its two antipode versions is essential in nowadays industries, in particular, in the pharma sector. Heterogeneous asymmetric catalysis represents the most appealing strategy because of its many advantages in terms of sustainability and recyclability as well as to the chiral multiplication effect where minor amounts of a chiral catalyst trigger the enantioselective production of vast amounts of chiral products, especially if such catalysts can be recycled. Enzymes represent the archetype of asymmetric catalysts, where the homochirality of *l*-amino acids that build their structure prompts the formation of asymmetric nanospaces that induce chirality during a catalytic reaction through stabilization of particular orientations of the reactant molecules.^1,2^ However, the exceptional asymmetric catalytic performance of enzymes is limited by several issues: (i) their homogeneous nature: they usually work in aqueous solution, and hence, their recyclability for reuse is difficult unless they are supported on heterogeneous systems, which can alter their enantioselective properties, (ii) their typically low activity in organic solvents (evolution has optimized their structure for performing reactions in the aqueous physiological media of cells), which limits their scope of reactions to those that can be performed in water, and (iii) the chiral limitation imposed by nature, where only one single enantiomer of a product (that dictated by the chiral selection of life) can be prepared. Hence, the development of catalytically active solids able to perform enantioselective heterogeneous catalytic operations under harsh conditions and in its two antipode versions remains as a formidable challenge in current research.^3,4^

In this context, the occurrence of chiral frameworks in microporous zeolitic materials provide an ideal opportunity to develop enantioselective catalytically active solids where the catalytic versatility, high hydrothermal stability, and shape-selectivity characteristic of zeolites would potentially drive the production of efficient asymmetric catalysts.^5−12^ Shape-selectivity in zeolite catalysts arises from their 3-dimensional periodic framework structures with pores and cavities of dimensions similar to those of the transforming molecules, which prompts the differentiation of reactants, transition states, or products within the zeolitic nanospace as a function of the geometric properties (size and shape) of the guest species allocated within.^13,14^ Chirality is also a geometric property, whose direct consequence is that the molecular shape of the two antipode versions of a chiral molecule are mirror images. Therefore, the phenomenon of chiral recognition, whereby chiral host systems recognize the handedness of chiral guest species interacting with, is directly connected to the different geometric match between diastereomeric host–guest chiral pairs. Hence, confinement in asymmetric nanospaces provided by chiral zeolite frameworks, in terms of both helicoidal channels and/or asymmetric cavities, could prompt the enantiodiscrimination of chiral guest species during a catalytic reaction, leading to heterogeneous stable asymmetric catalysts.

Although several chiral zeolites have been known for years,^6^ these have invariably crystallized as a mixture of both enantiomeric versions of their polymorphs, giving racemic solids with no enantioselective properties.^15^ Only in recent years has the enantio-enriched crystallization of chiral zeolites been possible through the use of chiral organic structure-directing agents (SDAs).^16−18^ These organic entities drive the crystallization of zeolite materials toward a particular framework type through a particular host–guest match with the size and shape of the void space of the zeolite that allocates the organic agents, thus imprinting the geometric properties of the latter in the zeolite void space.^19−21^ If a suitable host–guest match is established, a chiral organic SDA could favor the enantioselective crystallization of a chiral zeolite framework (enriched in one of its two enantiomorphic polymorphs), as was first demonstrated by Davis and co-workers for the medium-pore STW framework type.^7,16,22^ More recently, after a systematic study of the structure-directing effect of chiral organic cations built from commercially available enantiomerically pure (1*R*,2*S*)-ephedrine and (1*S*,2*S*)-pseudoephedrine alkaloids,^23−25^ we discovered GTM-3, a germanosilicate zeolite with the chiral -ITV framework structure,^17,26^ by using (1*S*,2*S*)-*N*,*N*-ethyl-methyl-pseudoephedrinium hydroxide (EMPS) as SDA,^27^ which contained extra-large and chiral pores that enabled to process very bulky molecules in an enantioselective fashion. Catalytic results clearly demonstrated the enantio-enrichment of the zeolite framework in one of its two antipode versions (*P*4_1_32 or *P*4_3_32) as a function of the handedness of the pseudoephedrine-derived organic cation [with (1*R*,2*R*) or (1*S*,2*S*) configuration]. Indeed, enantiomeric excesses up to ±51% have been achieved by using GTM-3 catalysts in the ring-opening of *trans*-stilbene oxide with 1-butanol.^28^ Interestingly, both from an application perspective and for the sake of a clear characterization of the chirality of the zeolite catalyst, both enantiomeric versions of the zeolite could be easily prepared from both enantiomers of pseudoephedrine, in contrast to enzymes or other catalysts derived from the chiral pool, where only one absolute configuration is available. Further research in our group with related pseudoephedrine-derived chiral SDAs led to the discovery of GTM-4, which contained the same -ITV framework structure, but where surprisingly the chirality was inversed after replacing the ethyl group in the SDA of GTM-3 by a benzyl group (*N*,*N*-benzyl-methyl-pseudoephedrinium).^29−31^ The counterintuitive observations made with these new chiral catalysts are directly connected to their novelty since the asymmetric host–guest chemistry developed within chiral zeolitic nanospaces, both during structure-direction by organic chiral cations along crystallization and during a catalytic reaction, is a very rare and novel phenomenon that has just started to be studied. Hence, the knowledge at the molecular level of how this chiral host–guest chemistry and the associated transfer of chirality takes place becomes crucial in order to understand and exploit their enantioselective properties. In this work, we perform a combined experimental and computational study in order to comprehend in depth the structural features that determine the asymmetric catalytic activity of GTM-3 chiral catalysts, in particular for the ring-opening of *trans*-stilbene oxide with 1-butanol, where notably high enantioselectivities have been found.^26^ Furthermore, this study is complemented with the determination of the absolute configuration of the zeolite catalysts by combining 3D electron diffraction (3DED) with Rietveld refinement methods in order to confirm the reaction mechanism proposed, to unravel the level of enantio-enrichment of these materials, and to understand the mechanism for the transfer of chirality from the organic SDA to the chiral zeolite polymorph.

## 2 Experimental Section

### 2.1 Synthesis of GTM-3 Catalysts

Synthesis of (1*R*,2*R*)-(−)- and (1*S*,2*S*)-(+)-*N*,*N*-ethyl-methyl-pseudoephedrinium hydroxide [hereafter referred to as (−)-EMPS and (+)-EMPS, respectively], used as SDAs for the synthesis of GTM-3 materials, was described in our previous work,^17^ starting from the corresponding commercially available chiral precursor, (1*R*,2*R*)-(−)-pseudoephedrine or (1*S*,2*S*)-(+)-pseudoephedrine (Sigma-Aldrich, 98%). Antipode GTM-3 zeolites were prepared following our previous recipe, from the corresponding (−)-EMPS or (+)-EMPS hydroxides.^17^ In order to avoid confusion with the *R*/*S* nomenclature of *trans*-stilbene oxide reactants and corresponding products, hereafter GTM-3 zeolite catalysts prepared from (1*R*,2*R*)-(−)- or (1*S*,2*S*)-(+)-EMPS cations will be referred to as (−)-GTM-3 and (+)-GTM-3, respectively; however, no relationship with optical rotation of the zeolite is meant.

### 2.2 Asymmetric Catalytic Activity of GTM-3

After calcination at 500 °C under air for 10 h, the (−)- and (+)-GTM-3 solids were used as acid-catalysts for the ring-opening of chiral *trans*-stilbene oxide with 1-butanol, which was reported in our previous work to show the highest enantioselectivity among a number of epoxides and alcohols of different size.^28^ Catalytic experiments were performed with 20 wt % of catalyst (with respect to *trans*-stilbene oxide) and with 1 mg/mL of racemic *trans*-stilbene oxide solution in 1-butanol. The reaction was carried out at room temperature under agitation, and aliquots were extracted at different time intervals. All manipulation of calcined GTM-3 samples was carried out under inert (N_2_) atmosphere (in a drybox) in order to avoid degradation of the framework. Evolution of the reaction to give the different products was monitored by HPLC with a chiral stationary phase (more details in the Supporting Information). Identification of the different enantiomers of the reactants and products (“*unlike*” and “*like*”) was accomplished by ring-opening of commercial enantiomerically pure (2*R*,3*R*)-2,3-diphenyloxirane ((*R*,*R*)-*trans*-stilbene oxide) (TRC) in 1-butanol homogeneously catalyzed with sulfuric acid, which gave (1*R*,2*S*)-2-butoxy-1,2-diphenyl-ethanol “*unlike*” product (via S_N_2) and (1*R*,2*R*)-2-butoxy-1,2-diphenyl-ethanol “*like*” product (via S_N_1).

### 2.3 Computational Study of the Reaction Mechanism

Due to the size and complexity of the catalytic system, a suitable large cluster consisting of 274 atoms was used as the -ITV model (with 73 T atoms), with a composition of Ge_3_Si_70_O_106_(OH)_15_(H_t65_), where interrupted Si positions were saturated with H atoms (H_t_); the composition of the *d4r* unit where the reaction takes place was Ge_3_Si_5_O_12_(OH)_2_. A preliminary set of calculations about the stability of Ge in the different positions of the *d4r* (there are two types of *d4r* units, one with two Q3 interrupted positions and another with only one), was performed in order to find the most appropriate configuration for the Ge atoms to analyze the reaction mechanism (in this case, using an appropriate cluster with 214 atoms, 62 T atoms). After the trends observed for the stability of Ge in the different positions, Ge atoms were located in both adjacent T7 positions, which correspond to the interrupted Q3 positions (with a dangling OH group), and in an adjacent T6 position (see Figures S1 and S2 in the Supporting Information) in the cluster Ge_3_Si_70_O_106_(OH)_15_(H_t65_); this model is large enough as to simulate the chiral framework structure around the main *d4r* unit that contains the active sites where the reaction will take place. In order to preserve the -ITV framework structure during the calculations, only atoms in the Ge-containing *d4r* unit and up to its second coordination shell, as well as all the terminal OH groups, were allowed to relax during geometry optimizations, keeping fixed the rest of the atoms. Calculations (geometry optimization and transition-state search calculations) were performed with the cluster model at the DFT + D level (PBEsol functional^32^ and the Tkatchenko and Scheffler dispersion term;^33^ this method has been shown to provide an excellent performance for modeling zeolite neutral systems).^34^ We used a numerical basis set (DNP+, double numerical plus polarization with diffuse functions) with the DMol3 code; this set of calculations allowed us to explore the vast and complex energy landscape of the reactions taking place within the catalyst. Calculations were performed with an -ITV cluster taken from the *P*4_1_32 polymorph. For the calculation of the transition states, an initial guess was estimated by selecting an appropriate interatomic distance as the reaction coordinate (of the bond being formed) and performing geometry-optimizations at different fixed intervals of such interatomic distance. Structure and activation energies of the transition states were then calculated using linear synchronous transit and quadratic synchronous transit methods, as implemented in DMol3. The structure of the transition state was confirmed by calculating vibrational frequencies to show the presence of a negative frequency corresponding to the atomic rearrangement taking place. Unless specified, energies are reported as relative internal energies [by subtracting the energy of the system with free (isolated) molecules]; all energies are given in kcal/mol. In selected cases, estimation of the free energies was carried out by adding the entropic contribution at a given temperature (298 K) after calculation of the Hessian.

### 2.4 Electron Diffraction to Determine the Absolute Configuration of GTM-3

3DED data were collected on a FEI Tecnai F20 TEM equipped with a DECTRIS QUADRO detector (512 × 512 pixels, pixel size:75 μm). A Fischione 2550 cryo transfer tomography holder (maximum tilt range: ±79°) was employed to load samples with liquid nitrogen, allowing for effective sample freezing to reduce the beam damage. Details are given in the results in Section 3.

## 3 Results

### 3.1 Experimental Study of the Asymmetric Catalytic Activity of GTM-3

**Scheme 1:**
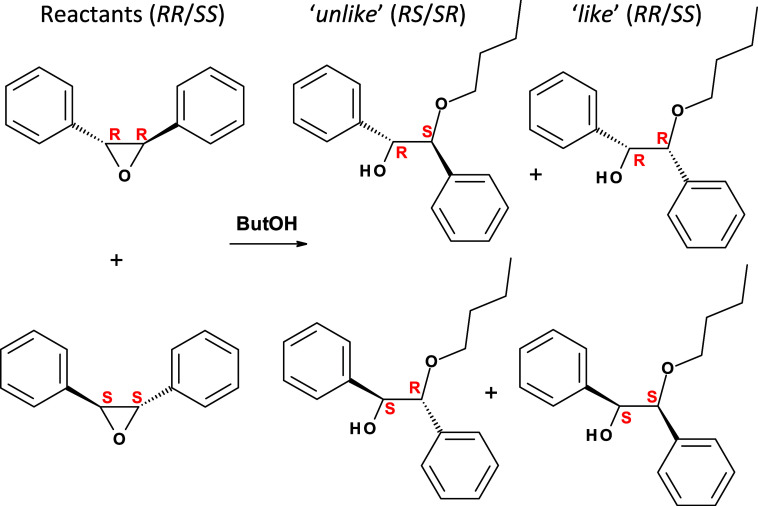
Ring-Opening Reaction of Chiral (1*R*,2*R*) (Top) or (1*S*,2*S*) (Bottom) *trans*-Stilbene Oxide with 1-Butanol, Yielding Major Chiral *Unlike*-Products (*R*,*S* + *S*,*R*) or Minor *Like*-Products (*R*,*R* + *S*,*S*); Species in Columns Are Enantiomers

**Figure 1:**
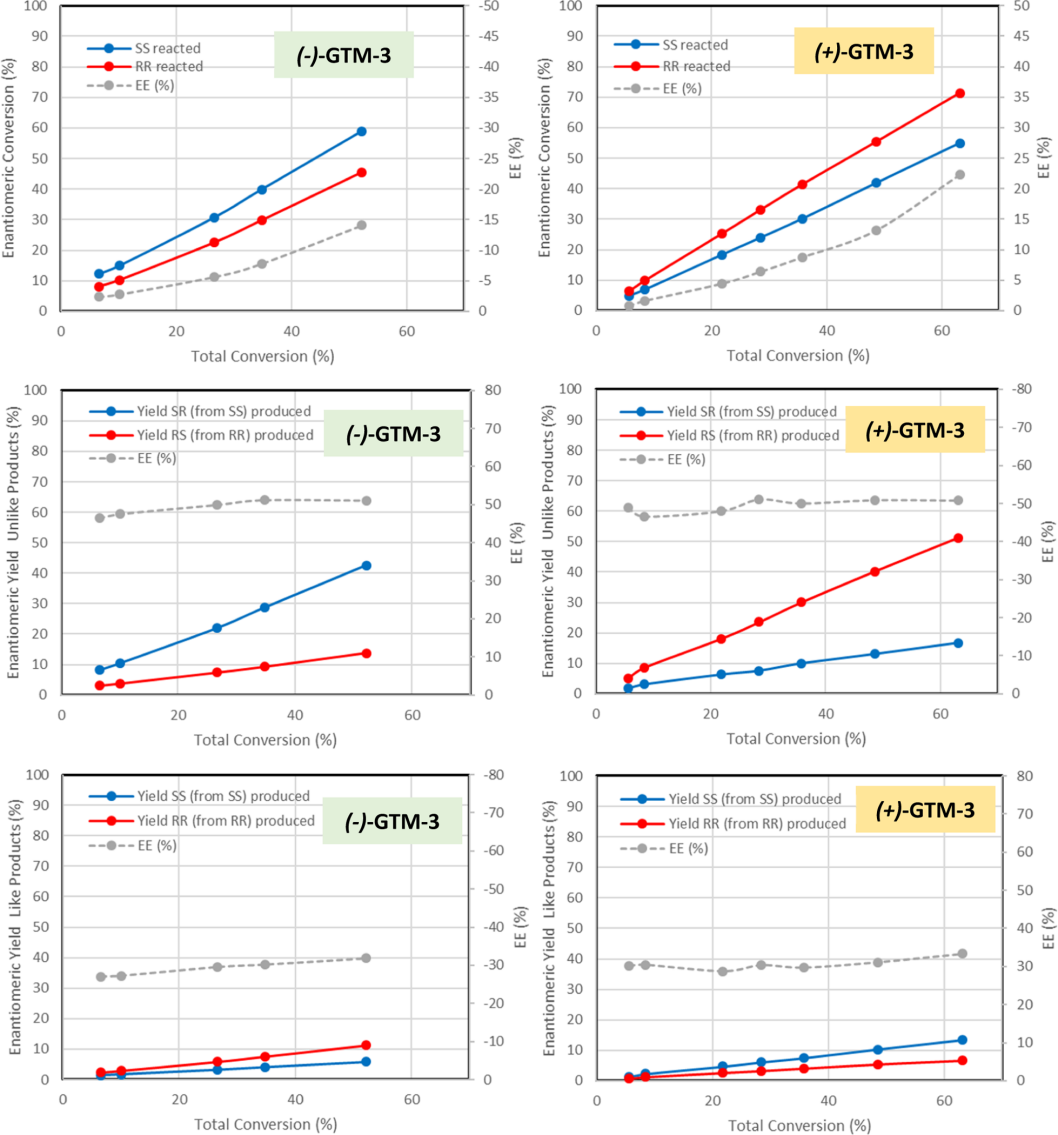
Asymmetric catalytic activity of (−)-GTM-3 (left) and (+)-GTM-3 (right) catalysts and corresponding enantiomeric excesses (*ee*). Top: conversion of each enantiomer of *trans*-stilbene oxide (*RR*/*SS*); middle: yield of *unlike* products (*RS*/*SR*); bottom: yield of *like* products (*RR*/*SS*). In all cases, enantiomeric conversions and yields (blue and red lines) refer to the left axis, and *ee* values to the right axis [note that the right axis for *ee*’s are inverted for (−)- and (+)-GTM-3 catalysts].

Hereafter, (2*R*,3*R*)-2,3-diphenyloxirane and (2*S*,3*S*)-2,3-diphenyloxirane will be referred to as *RR*-TSO and *SS*-TSO reactants, (1*R*,2*S*)-2-butoxy-1,2-diphenyl-ethanol and (1*S*,2*R*)-2-butoxy-1,2-diphenyl-ethanol will be referred to as *RS*-*unlike* and *SR*-*unlike* products, and (1*R*,2*R*)-2-butoxy-1,2-diphenyl-ethanol and (1*S*,2*S*)-2-butoxy-1,2-diphenyl-ethanol as *RR*-*like* and *SS*-*like* products, respectively (see Scheme 1). First, we identified the absolute configuration of each enantiomer of reactants (*RR*-TSO and *SS*-TSO), *unlike* products (with inversion of configuration, giving *RS*-*unlike* from *RR*-TSO and *SR*-*unlike* from *SS*-TSO, both of which are produced mainly via an S_N_2 mechanism), and *like* products (with retention of configuration, giving *RR*-*like* product from *RR*-TSO reactant and *SS*-*like* from *SS*-TSO, produced via an S_N_1-like mechanism) (see Scheme 1) by comparison with the reaction carried out with enantiomerically pure *RR*-TSO with sulfuric acid. Once the different enantiomers for each species were identified, we performed a complete study of the asymmetric catalytic activity of (−)-GTM-3 [prepared from (−)-(*RR*)-EMPS as SDA] and of (+)-GTM-3 [prepared from (+)-(*SS*)-EMPS]. Figure 1 shows the conversion of each TSO enantiomer (top) and the yield of the *unlike* (middle) and *like* (bottom) products as well as the corresponding enantiomeric excesses (*ee*, gray lines) when using antipode (−)-GTM-3 (left) or (+)-GTM-3 (right) catalysts. First of all, an exactly opposite behavior when using both GTM-3 catalysts prepared with opposite enantiomers of EMPS in the synthesis (compare Figure 1, left and right) can be noticed, which clearly demonstrates the opposite chiral enantio-enrichment of both zeolite catalysts. Results show that the *SS*-TSO reactant converts faster with the (−)-GTM-3 catalyst [and *RR*-TSO with the (+)-GTM-3 catalyst], with the enantiomeric excess of the remaining reactants progressively growing as the reaction proceeds, as typical for this type of reactions carried out with racemic mixtures of chiral reactants. In line with this, the higher conversion of *SS*-TSO with (−)-GTM-3 involves a higher production of *SR*-*unlike* product via the S_N_2 mechanism (Figure 1-middle), with a stable *ee* value around +50% all along the reaction, and the opposite is found for the (+)-GTM-3 catalyst, with a higher production of *RS*-*unlike* product (*ee* around −50%). An opposite behavior is found for the *like* products that are produced via an S_N_1-like mechanism, with retention of configuration (Figure 1, bottom): in this case, the (−)-GTM-3 catalyst promotes a higher production of the *RR*-*like* product (coming from *RR*-TSO), and as always, exactly the opposite behavior is found for the (+)-GTM-3 catalyst, with a higher production of *SS*-*like* product (from *SS*-TSO), giving in both cases *ee* values around ±30%. These observations indicate an opposite behavior for both types of products (*unlike* and *like*) and consequently, for both types of mechanisms (S_N_2 for *unlike* and S_N_1 for *like*): (−)-GTM-3 favors the formation of the most abundant *SR*-*unlike* products from *SS*-TSO, which reacts faster, and simultaneously the formation of the minor *RR*-*like* products from the antipode *RR*-TSO reactant, and the opposite for (+)-GTM-3.

**Figure 2:**
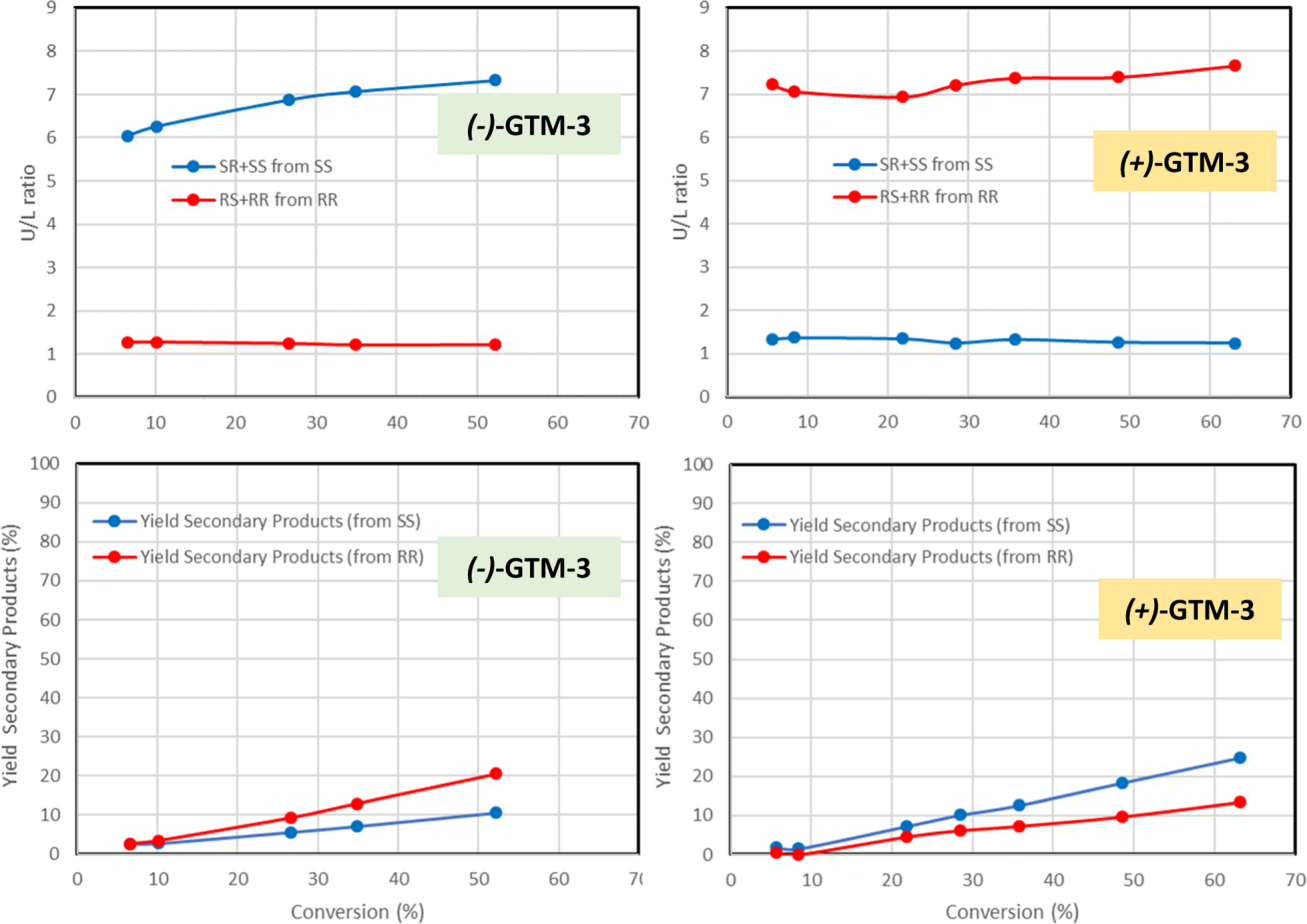
*Unlike*/*like* products ratio (top) and yield of secondary products (bottom) for (−)-GTM-3 (left) and (+)-GTM-3 (right) catalysts.

Figure 2 shows the *unlike*/*like* (*U*/*L*) ratio of products derived from *SS*-TSO (blue line, *SR*-*unlike*/*SS*-*like*) and *RR*-TSO (red line, *RS*-*unlike*/*RR*-*like*) (top). When (−)-GTM-3 is used as catalyst, where *SS*-TSO reacted faster, giving preferentially *SR-unlike* products, a much higher *U/L* ratio is observed for products derived from *SS*-TSO (blue line, around 7) than for those derived from *RR*-TSO (red line, slightly higher than 1), and exactly the opposite is found for the (+)-GTM-3 catalyst (Figure 2, right), with a much higher *U/L* ratio for products obtained from the *RR*-TSO reactant (red line) that is transformed faster than for those of the less reactive *SS*-TSO (blue line), giving values very similar to those of the antipode (−)-catalyst. These results indicate that the TSO enantiomer that reacts faster on each catalyst is converted mostly through the S_N_2 mechanism to give the *unlike* product with inversion of configuration, while the less reactive TSO enantiomer proceeds mostly through the S_N_1-like mechanism that produces a mixture of *unlike* (with inversion) and *like* (with retention of configuration) products. We then determined the formation of secondary products (defined as the sum of products other than those of the *unlike* and *like* configuration); these products were calculated as the difference between the reacted TSO (for each enantiomer) and the sum of the *unlike* and *like* products (from each TSO enantiomer) (Figure 2, bottom). Interestingly, a clear trend is found: in the case of the (−)-GTM-3-catalyzed system, a notably higher ratio for the formation of secondary products is observed for those derived from the *RR*-TSO reactant (red line), which reacts mostly through the S_N_1 mechanism, and the opposite is found for the (+)-GTM-3 catalyst, with a higher formation of secondary products from the less reactive *SS*-TSO reactant (blue line). These results suggest that the formation of secondary products is connected to the formation of the *like* chiral products via an S_N_1-like mechanism. We were able to identify diphenylacetaldehyde as the major component of those secondary products (∼40%) as well as of benzophenone (although in a very minor proportion, less than 1%); in addition, a pair of what seemed to be enantiomeric quasi-racemic products and another product were observed.

**Scheme 2:**
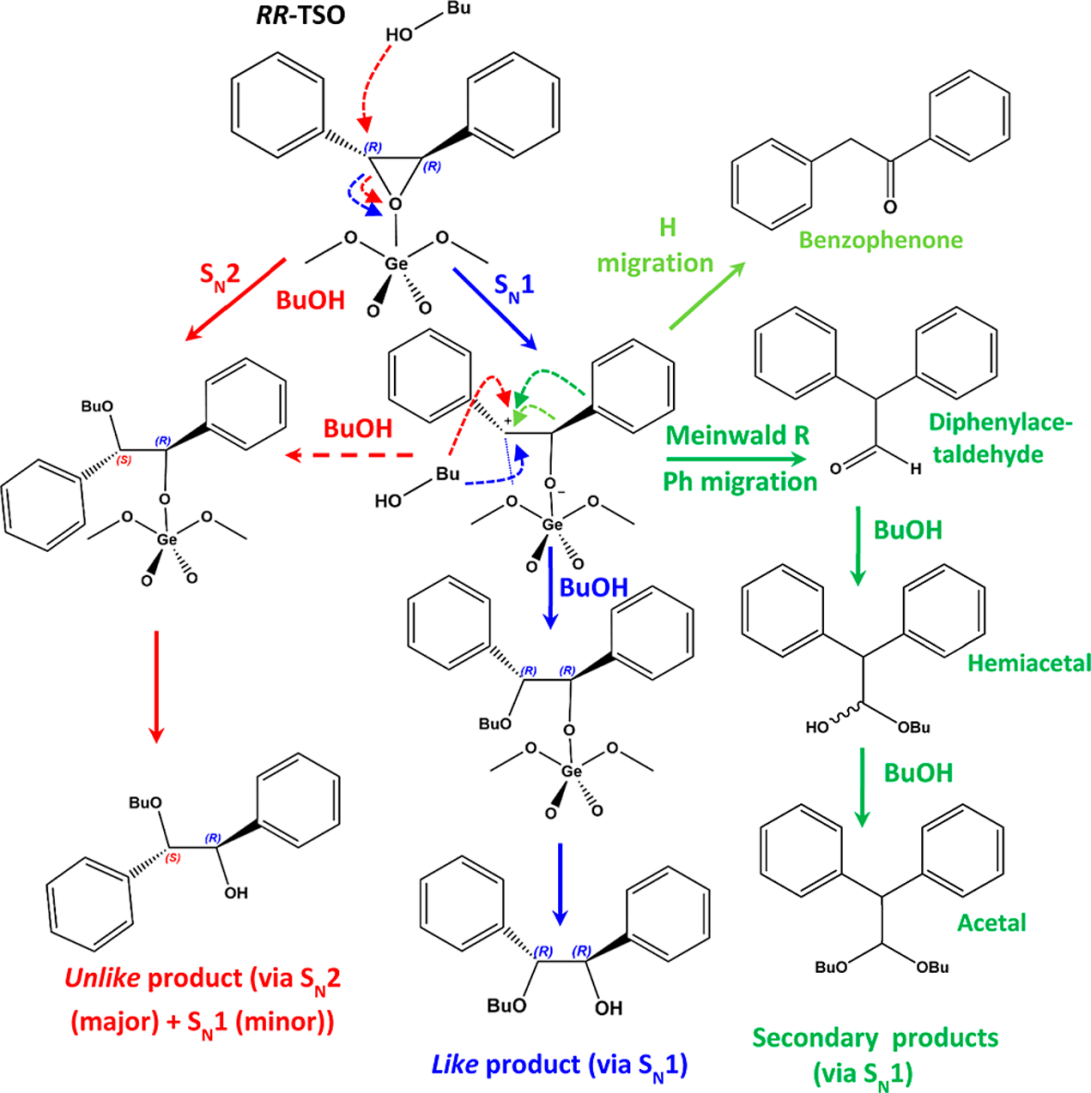
Reaction Mechanism Proposed for the Transformation of *RR*-TSO in the Presence of GTM-3 Catalysts via S_N_2 or S_N_1 Routes (the Analogue Mechanism for *SS*-TSO Is Shown in Scheme S1 in the Supporting Information)

Based on all these results, we propose the following reaction mechanism for the asymmetric transformation of TSO in the presence of 1-butanol catalyzed by GTM-3 chiral catalysts (Scheme 2); the mechanism is displayed for the *RR*-TSO reactant but is the same for *SS*-TSO (shown in Scheme S1 in the Supporting Information). *RR*-TSO is activated by Ge and then transforms into the corresponding *RS-unlike* product via an S_N_2 route that occurs through an attack of butanol from the opposite side of the oxirane ring, resulting in an inversion of configuration (red arrows, S_N_2). Alternatively, *RR*-TSO can form the corresponding carbocation-like species by opening the oxirane ring through an S_N_1-like mechanism (blue S_N_1 route). Such carbocation could then be attacked by butanol through either side, with an attack from the same side as the O giving the *RR*-*like* product (with retention of configuration, blue arrows) or an attack from the opposite side giving again the *RS-unlike* product (dashed red route). The carbocation could also transform through a *Meinwald* rearrangement reaction by migration of the phenyl ring to the positive C atom to give diphenylacetaldehyde (green route) or alternatively through H-migration to give benzophenone (light green route). Diphenylacetaldehyde can then be further transformed in the presence of butanol and with acid catalysis to give the corresponding hemiacetal and acetal products; all these represent the secondary products (green routes).

**Figure 3:**
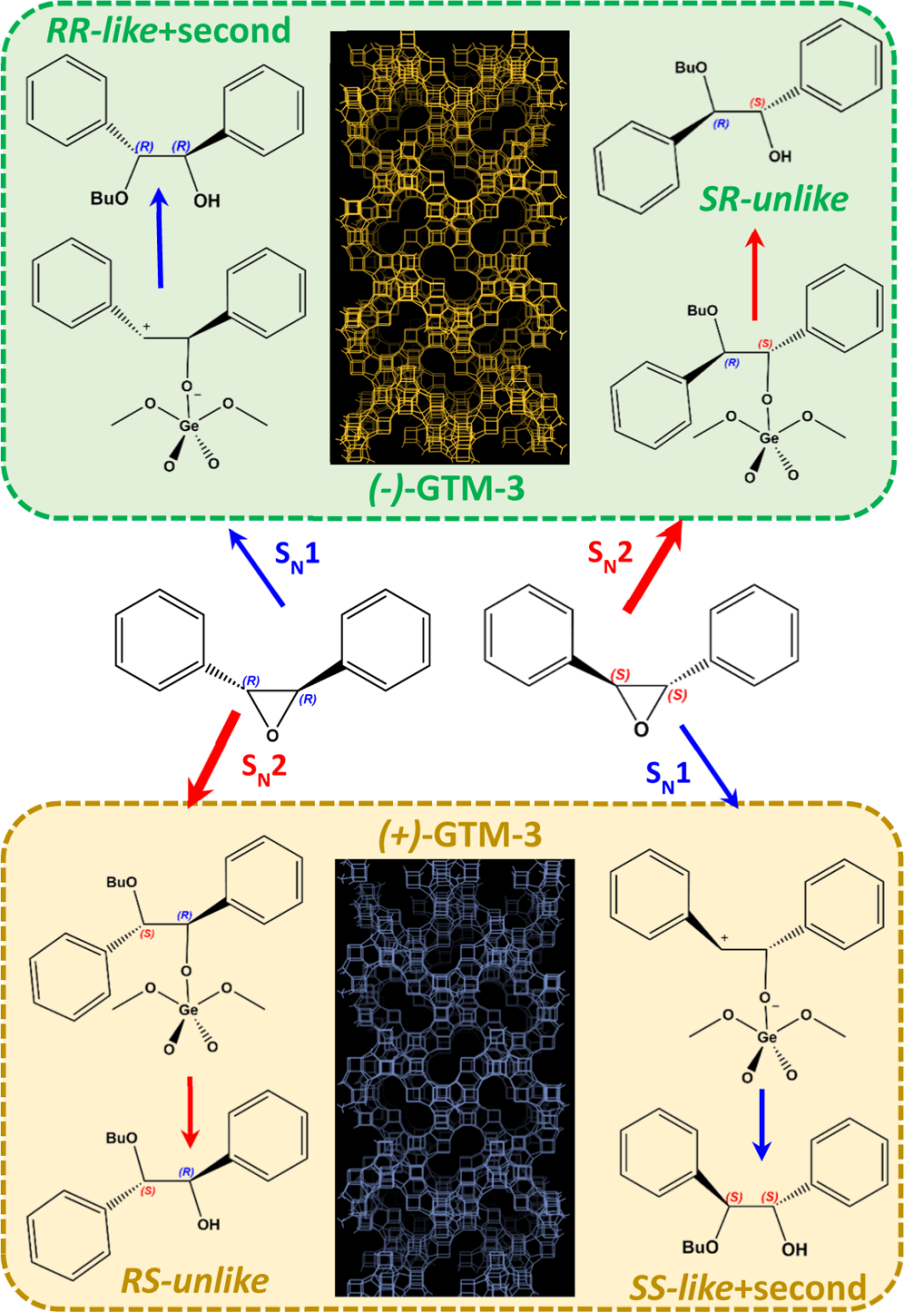
Prevalence of S_N_2 and S_N_1 mechanistic routes to give the chiral products from racemic TSO using (−)-GTM-3 (green) and (+)-GTM-3 (yellow) catalysts.

Thus, in accordance with our observations, both secondary and chiral *like* products are connected and produced by the S_N_1-like mechanism, while the *unlike* products are produced mainly through the S_N_2 mechanism, though a certain amount should also be formed through the S_N_1 mechanism (dashed red route). In this way, the identification of the different enantiomers of each chiral species allowed us to determine the prevalence of S_N_2 and S_N_1 mechanistic routes to give the chiral products from racemic TSO using antipode (−)-GTM-3 (green) and (+)-GTM-3 (yellow) catalysts (Figure 3); in the presence of the (−)-GTM-3 catalyst, *SS*-TSO reacts faster, giving mainly *SR-unlike* products through the S_N_2 route, while *RR*-TSO reacts at a slower rate, and in this case mainly through the S_N_1 route to give preferentially the *like* and secondary products, and the opposite occurs for the (+)-GTM-3 catalyst, where *RR*-TSO reacts faster to give the *RS-unlike* products and *SS*-TSO gives mainly the *SS-like* and secondary products. These results suggest that the origin of the enantioselectivity of GTM-3 catalysts in this particular reaction is associated with a different mechanistic route for the transformation of each TSO enantiomer with butanol into the chiral products as a function of the catalyst handedness.

Worth is noting a final remark about the *unlike* products, which under these conditions gave an *ee* around 50%. Part (if not most) of the less preferential enantiomer for the S_N_2 route [*RS-unlike* for the (−)-GTM-3 catalyst and *SR-unlike* for the (+)-GTM-3 catalyst] should be produced via S_N_1 (dashed red route in Scheme 2 and Supporting Information). Indeed, a similar amount of *like* and *unlike* products of the less reactive TSO enantiomer (U/L ratio around 1.2) is observed (see Figure S3 in the Supporting Information), which might point to a large fraction of the less reactive *unlike* product being produced via S_N_1. This brings as a consequence that the enantioselectivity observed for the S_N_2 route should be even higher (if we consider exclusively the enantioselectivity of this S_N_2 route) than that apparently observed (∼50%) since part (if not most) of the less reactive *unlike* product should be produced by other routes.

Finally, in order to gain additional insights for the enantioselectivity associated with the S_N_1 route, we made TSO to react within GTM-3 catalysts but in the absence of the alcohol nucleophile (using toluene as solvent): under these conditions, only the *Meinwald* rearrangement reaction can take place to give diphenylacetaldehyde. By plotting the conversion of each enantiomer of TSO, we could estimate whether GTM-3 catalysts could prompt some enantioselectivity along this route. Interestingly, enantioselectivity appeared (see *ee* of the remaining reactants in Figure S4 in the Supporting Information): results showed a stronger transformation into diphenylacetaldehyde for *RR*-TSO in (−)-GTM-3 [and for *SS*-TSO in (+)-GTM-3]. Interestingly, this enantioselectivity represents the opposite behavior from that observed for the S_N_2 route to give the *unlike* products, where *SS*-TSO reacted faster in (−)-GTM-3, and hence indicates that both reaction mechanisms display opposite enantioselectivities under these chiral catalysts.

### 3.2 Computational Study of the Chiral Reaction Mechanism of GTM-3

After this comprehensive experimental study of the reaction mechanism within chiral GTM-3 catalysts, we performed a DFT + D study of the possible reaction pathways (as far as our chemical intuition could go) for the transformation of TSO with 1-butanol. We first analyzed the stability of Ge clusters embedded within the -ITV framework, then the potential activation of TSO on Ge sites, and finally the addition of butanol to TSO and transformation into the *unlike* products in the Ge-containing -ITV cluster models. All of these calculations were performed with the -ITV cluster derived from the *P*4_1_32 polymorph.

#### 3.2.1 Stability of the Ge Clusters

First of all, we analyzed the stability for the localization of Ge in different positions of the -ITV framework. There are 10 crystallographic T positions: T4, T5, T6, and T7 belong to a *d4r* unit with two interrupted Q3 positions terminating with dangling OH groups in the two T7 sites (there are 12 of these rings per unit cell, hereafter referred to as *d4r*-A); T1, T2, T8, and T9 belong to another *d4r* unit with only one interrupted position in the T8 site (there are 8 of these rings per unit cell, hereafter referred to as *d4r*-B); and finally, T3 and T10 are positions not belonging to *d4r* units but to a *lau* cage connecting the *d4r* (see Figure S2 in the Supporting Information). For these calculations, we used a Si_62_O_104_(OH)_8_H_t32_ cluster, introducing 1, 2, or 3 Ge (replacing the corresponding Si atom) and relaxing the atomic coordinates of the *d4r* units under study as well as up to its second coordination shell.

When 1 Ge is introduced, a clear higher stability was found for Ge in the TOH interrupted positions, both T7 (in *d4r*-A) and T8 (in *d4r*-B), with relative energies (with respect to the most stable case) lower than 0.5 kcal/mol (Table S1 in the Supporting Information). Positions T6 and T5 (in *d4r*-A) and T1 (in *d4r*-B) are next in stability with relative energies between 1 and 2 kcal/mol. Positions T4 (in *d4r*-A) and T2 and T9 (in *d4r*-B) are less stable for Ge (2–3 kcal/mol); finally, Ge in positions of the *lau* cage (not belonging to *d4r* units) is much less favorable (3–6 kcal/mol), as expected. This clearly indicates a preferential occupation of the interrupted TOH sites of the *d4r* units by Ge, in agreement with previous observations.^26^

The most stable incorporation of a second Ge in *d4r*-A corresponds to both clustered Ge occupying adjacent T7 interrupted positions, followed by clustered Ge dimers in T7 and adjacent T6 positions (with relative energies between 1 and 1.5 kcal/mol); these results are in line with the higher stability of T7 followed by T6 (Table S1) and the trend of Ge to cluster through formation of Ge–O–Ge bonds;^35^ our results show that, in general, clustered Ge is preferred to isolated Ge sites. Accordingly, the most stable incorporation of 3 Ge atoms in *d4r*-A corresponds to the occupation of both T7 interrupted positions and one adjacent T6 position, forming a cluster of 3 Ge atoms (Figure S5, top, in the Supporting Information). Incorporation of 3 Ge per *d4r* unit (and no Ge on T3/T10 positions not belonging to *d4r*) would give a Si/Ge ratio of 2.2, which is in line with the experimental Si/Ge ratios of GTM-3 determined by EDX (1.9). Hence, this represents the Ge-containing *d4r*-A system that will constitute the active site, where the mechanistic computational study will be performed (see below). On the other hand, the most stable incorporation of 2 Ge in *d4r*-B units involves the location in T8 interrupted and adjacent T1 positions, in line with their higher stability observed for 1Ge systems (Table S1). Similarly, the incorporation of 3 Ge in *d4r*-B units is favored for Ge clusters in T8 interrupted and both adjacent T1 positions (Figure S5, bottom). Nonetheless, a higher stability for 3 Ge-clusters is observed in *d4r*-A units, probably due to the occurrence of two interrupted (T7) positions (instead of one in *d4r*-B) that are favored for Ge.

#### 3.2.2 Adsorption and Activation of *trans*-Stilbene Oxide

**Figure 4:**
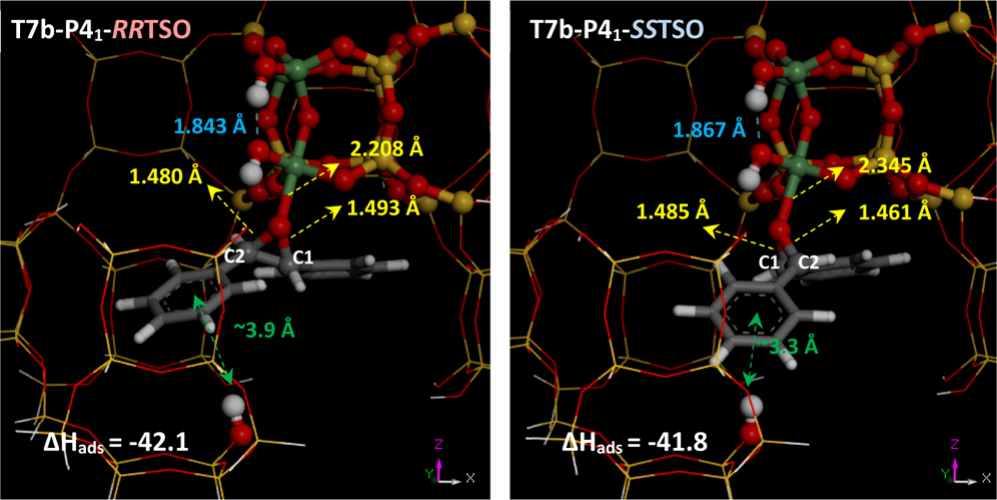
Formation of Ge(T7)···TSO complexes with *RR*-TSO (left) and *SS*-TSO (right). Hereafter, O, H, C, Ge, and Si atoms are displayed in red, white, gray, green, and yellow, respectively.

The next step was to study the adsorption and potential activation of TSO in its two enantiomeric forms. Of all the possible modes of adsorption and orientations, two were found as the most favorable ones. One involved the formation of an H-bond from the framework OH of the interrupted Ge7 position to the O atom of TSO, giving adsorption (internal) energies (Δ*E*) of −47.9 kcal/mol for *RR*-TSO and −49.3 kcal/mol for *SS*-TSO (Figure S6 in the Supporting Information); in this case, no notable activation of the oxirane ring (estimated as the elongation of the C–O bond) was observed (C–O distances were 1.45/1.46 Å, being 1.44/1.44 Å in free TSO). Because of its Lewis acidity, the other mode of adsorption involves coordination with Ge; in this case, the most stable complex of TSO that we found is formed with Ge in the T7 position, which is probably related to its higher flexibility because of the presence of the interrupted OH group. Figure 4 displays the structure of *RR*-TSO (left) and *SS*-TSO (right) complexes with Ge, giving similar adsorption internal energies of −42.1 and −41.8 kcal/mol, respectively; incorporation of the entropic term gives free energies (at 298 K, Δ*G*(298)) of −40.7 and −38.9 kcal/mol for *RR*-TSO and *SS*-TSO, respectively. Interestingly, the rearrangement of Ge to alter its coordination environment from tetrahedral (*N* = 4) to trigonal bipyramid (*N* = 5) upon binding of TSO prompts an approach of the two dangling OH groups associated with Ge(T7) interrupted positions that promotes the development of a new intraframework H-bond between them (Figure 4, dashed blue line), promoting an additional stabilization to these Ge complexes [compare with free Ge(T7)OH in Figure S6]. Worth noting is the different orientation of the oxirane ring in both TSO enantiomers, which sites are parallel to the *xz* (*RR*-TSO) or *yz* (*SS*-TSO) planes, as a result of the different absolute configuration of the oxirane C atoms (see Figure S7 in the Supporting Information for more details). In this case, coordination through the oxirane atom promotes the activation of the C–O bonds (C–O is elongated, with distances of 1.480 and 1.493 Å for *RR*-TSO and 1.461 and 1.485 Å for *SS*-TSO). In both cases, one of the two C–O bonds is more strongly activated (the one closer to the *d4r* unit, which will be referred to as C1). Worth remarking is that the formation of these complexes in their particular orientation is strongly favored by the development of interactions of the phenyl rings with a nearby OH group of another *d4r* unit (green arrows in Figure 4). In sum, both the asymmetric nature of the -ITV cavities with the two adjacent Ge(T7)OH groups (in *d4r*-A units) that enables the development of stabilizing intraframework H-bonds upon coordination of TSO and the presence of nearby OH interrupted framework groups in nearby *d4r* units are responsible for the different orientation of the TSO enantiomers as a function of their absolute configuration, where a cooperative mechanism involving several chemical species is at work. Interestingly, a close host–guest size match between the TSO and the -ITV cavities can be observed (see Figure S7-bottom).

Alternatively, formation of an analogue Ge···TSO complex but with Ge(T6) in the same type of *d4r*-A unit is less favorable due to its lower flexibility (no interrupted OH position associated). Similarly, our results suggest that the formation of these preactivated complexes (with elongated C–O bonds) is less favorable for Ge in the interrupted T8 position of the other type of *d4r*-B units likely because in this case, no intraframework H-bond can be developed (there is only one T8 interrupted position per *d4r-B* unit). Hence, hereafter, only *d4r-A* units will be studied. On the other hand, prior activation of butanol to give bound butoxide species that would react with free TSO is not a favorable pathway for the S_N_2 route due to the bulky nature of TSO that hinders an approximation of the oxirane ring (from the opposite side to O) to the bound O of activated butoxide.

#### 3.2.3 S_N_2 Route: Attack of Butanol to Give *Unlike* Products

**Figure 5:**
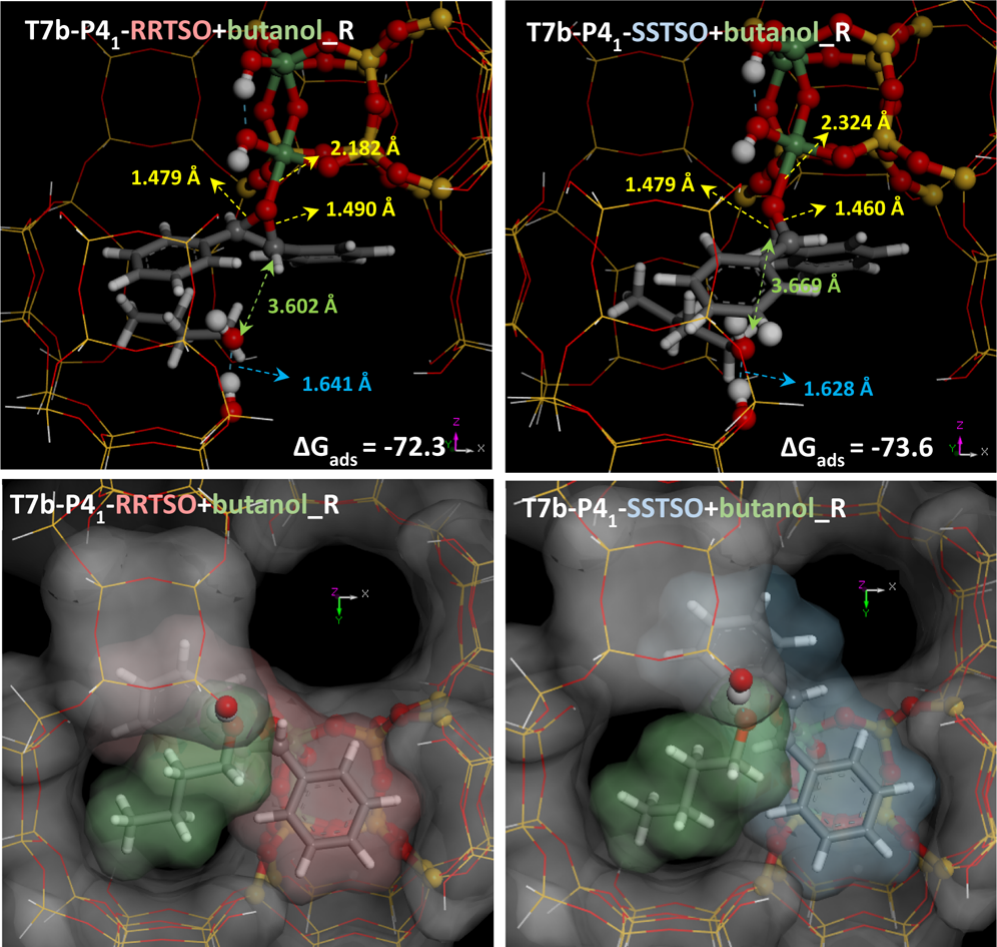
Initial orientation for the attack of butanol to coordinated TSO through the S_N_2 mechanism; two views are shown (top and bottom); Connolly surfaces (white for -ITV, red for *RR*-TSO, blue for *SS*-TSO, and green for butanol) are displayed (bottom) in order to highlight the host–guest size match between reactants and the -ITV cavity.

Once the TSO reactant molecules are activated through coordination with Ge(T7), the next step is the attack of a nearby butanol molecule from the opposite side to the oxirane ring through an S_N_2 route. We first looked for the most favorable approach of a butanol molecule to preactivated TSO. Given the orientation of TSO bound to Ge(T7), the most stable approach for butanol that we found was from below, where butanol was stabilized through an H-bond with a framework OH group (1.64–1.63 Å) (Figure 5, top); butanol was further stabilized by nonbonded interactions with the walls of the -ITV cavities, and indeed a close host–guest size match was found for this epoxide/alcohol system within the -ITV chiral cavities (see Figure 5, bottom). Calculation of the free energies of these systems showed a slightly higher stability for the *SS*-TSO/butanol host–guest complex [Δ*G*(298 K) = −73.6 kcal/mol] than for RR-TSO/butanol [Δ*G*(298 K) = −72.3 kcal/mol]. Our study suggests that an important role of the -ITV framework is to promote particular orientations and a singular approach of the reactants as well as the preactivation of the oxirane C–O bonds to promote the addition of butanol, which was more favorable for C1; this is reminiscent of the action of enzymes.

**Figure 6:**
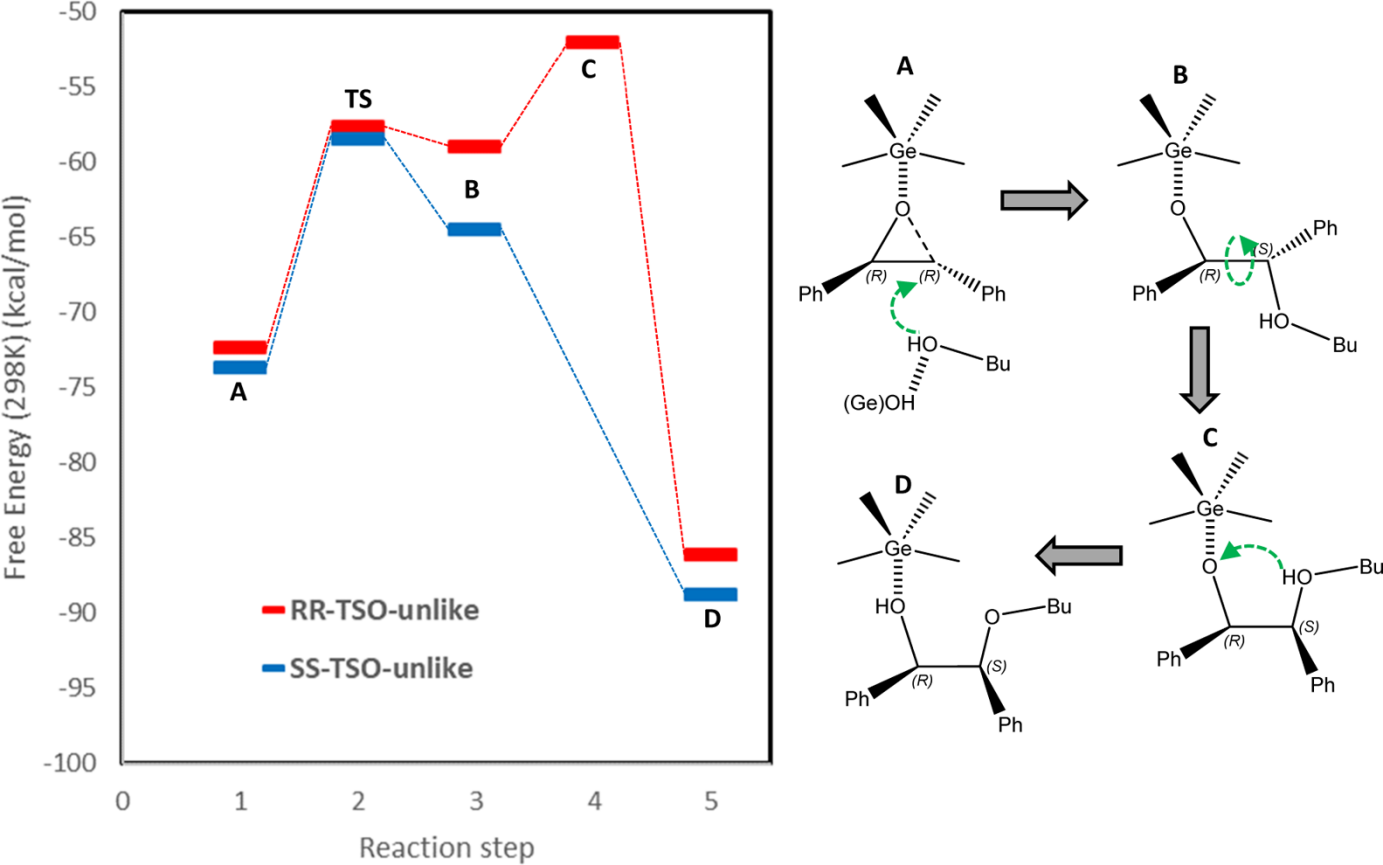
Free energy profile (at 298 K, left) and scheme of the reaction mechanism (right) for the S_N_2 addition of butanol to the oxirane ring; the scheme is displayed for the transformation of *RR*-TSO on *RS*-*unlike* products, but the same would occur for *SS*-TSO.

TS search engines were then employed to find the transition state for such a butanol addition. Figure 6 displays the free energy profile for the addition of butanol to TSO and the corresponding reaction scheme. We focus on the addition of butanol to C1 (see Figure 4), which was more favorable; this promotes the opening of the oxirane ring through breaking of the C1···O bond (steps A → B in Figure 6). Free energy results show a similar activation barrier of ∼15–16 kcal/mol for both enantiomers, but the reaction free energy is more favorable for *SS*-TSO (Δ*G*_r_ = −64.4) than for *RR*-TSO (Δ*G*_r_ = −58.9). The structures of the TS and of the product upon addition of butanol (B in Figure 6) are shown in Figure S8 in the Supporting Information. While both TSs are stabilized to a similar extent, the geometry of the *P*4_1_32 -ITV cavities displays the appropriate size to stabilize preferentially the *SS*-TSO···BuOH product complex (B); such higher stability is likely at least in part provided by the interaction of the nearby OH group to the phenyl ring in the *SS*-TSO···BuOH complex (Figure S8, bottom, blue arrow).

**Figure 7:**
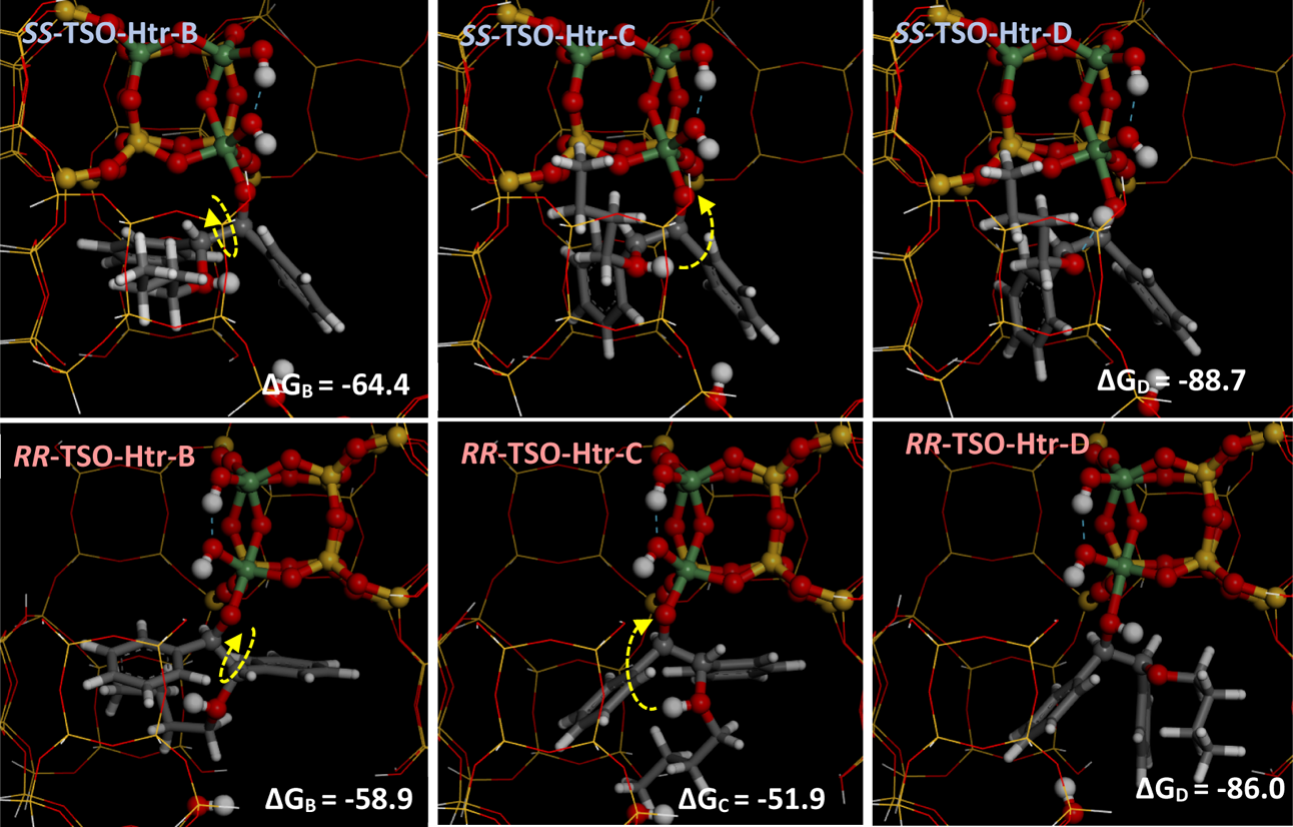
Structure of key species for the final H-transfer from *SS*-TSO···BuOH to give the final *unlike* product (1*S*,2*R*)-2-butoxy-1,2-diphenyl-ethanol (top) and from *RR*-TSO···BuOH to give the final *unlike* product (1*R*,2*S*)-2-butoxy-1,2-diphenyl-ethanol (bottom). Note the different orientation for *SS*- (top) and *RR*- (bottom) cases.

Formation of the *unlike* products requires a subsequent transfer of the H atom from the hydroxyl-group of added butanol to the O atom of TSO (step B → D in Figure 6); since the oxirane ring is already dissociated, this can take place just by rotating the substituent with the added butanol around the C1–C2 bond (step B → C) in order to approach both O-moieties, which triggers a barrier-less H-transfer from the butanol hydroxyl-group to O (step C → D). This C–C bond rotation process takes place spontaneously for the *SS*-TSO···BuOH intermediate without any energy barrier because of a proper host–guest chemistry for such rotation to occur (Figure 6-left, step B → D). Once the C–C bond rotates beyond a certain threshold, then both O atoms are close enough (O···O distance below 3.2 Å) so as to allow a barrierless H-transfer to give the final *unlike* products where C2 has inverted its absolute configuration ((1*S*,2*R*)-2-butoxy-1,2-diphenyl-ethanol) (Figure 7-top), being a barrierless and very favorable process (Δ*G*(298) = −88.7). In contrast, the analogous C–C bond rotation is sterically hindered for *RR*-TSO···BuOH (Figure 7-bottom). Because of the different orientation of the complex formed with Ge, such C–C bond rotation involves a complete turn of the butanol group added that along the way loses its interaction with the framework walls (Figure 7, bottom), thus resulting in a loss of stabilization that prompts a barrier for such a rotation of 7 kcal/mol. As in the previous case, once the C–C bond turns, then both O atoms approach so as to enable a barrierless H-transfer to give the final (1*R*,2*S*)-2-butoxy-1,2-diphenyl-ethanol *unlike* product (Δ*G* = −86.0).

The same set of calculations was also performed for the attack of butanol to the less activated C atom (C2), but a rather less favorable free energy profile was found for the addition of butanol to both *RR*-TSO and *SS*-TSO cases to break the oxirane ring (see Figure S9 in the Supporting Information), suggesting the preference for the S_N_2 mechanism through the addition of butanol to C1, which indeed is the most activated C atom of the oxirane ring upon adsorption on Ge. In any case, also through this mechanism, *SS*-TSO is favored with respect to *RR*-TSO.

Our computational reaction model predicts a more favorable S_N_2 synthetic route for the *SS*-TSO reactant to give *SR*-unlike products with GTM-3 catalysts in the *P*4_1_32 polymorph. If we now compare with our experimental catalytic results, we observe a higher formation of *SR*-unlike products for the (−)-GTM-3 catalyst (Figure 1), which was prepared from *RR*-(−)-EMPS SDA; this would predict that the -ITV framework obtained with *RR*-EMPS as SDA should be enriched in the *P*4_1_32 polymorph and the opposite for *SS*-EMPS, that should lead to -ITV enriched in the *P*4_3_32 space group.

In sum, our computational and experimental reaction results indicate that *SS*-TSO reacts more favorably in *P*4_1_32 enantiomorphic polymorph (produced from *RR*-EMPS), giving preferentially *SR*-*unlike* products, while *RR*-TSO formation of the corresponding *RS*-*unlike* products is strongly hindered because of a steric impediment to the rotation of the butanol substituent to enable the final H-transfer, and hence, this enantiomer undergoes instead a S_N_1 route to give the *RR*-*like* and secondary products, which is indeed favored for this enantiomer, and the opposite occurs for *P*4_3_32 (directed by *SS*-EMPS), which gives mainly *RS*-*unlike* products and *SS*-*like* products. Hence, we conclude that, at least for this particular reaction, the origin of the strong enantioselectivity observed for our GTM-3 catalysts is associated with the -ITV asymmetric cavities and the catalytic activity of the GeOH active sites in interrupted T7 positions, which prompts a particular orientation of TSO reactant molecules when they form the corresponding complex with Ge(T7) sites that is stabilized by the formation of an intraframework H-bond between both adjacent Ge(T7)OH sites and the interaction with nearby TOH groups in the cavity. Such a specific orientation of the TSO enantiomers within the confined space of the -ITV cavities makes them display strongly restricted translational and orientational degrees of freedom (in contrast to what would happen in a solvent), thus prompting an enantioselective discrimination during the course of the reaction that is mainly determined by the facility of the C–C bond rotation of the butanol moiety to enable the final H-transfer reaction. Such particular enantioselective behavior is maximized when a host–guest chiral size match occurs, which in our case is met for TSO and butanol reactants confined within the -ITV cavities (Figures 5 and S7).

### 3.3 Determination of the Absolute Configuration of GTM-3 Catalysts by 3D-Electron Diffraction

We finally wanted to confirm the absolute configuration of the GTM-3 catalysts predicted by the computational model. The framework structure of GTM-3 was determined directly by 3DED using the ShelXle software package, but the absolute structure determination is still a challenge. For GTM-3, the crystal size was only hundreds of nanometers, which made it impossible to obtain the absolute structure by single X-ray diffraction. For electron diffraction, electrons encounter multiple scattering when they interact with matters, which results in nonlinear deviations from kinematical intensities in electron diffraction patterns, what is known as dynamical effects. Because of this, the determination of the absolute structure is not a routine approach. Thanks to the dynamical diffraction theory, Klar and co-workers developed a method for the routine determination of absolute structures by using 3DED data.^36^ By using this method, we analyzed 15 crystals of (+)-GTM-3 [prepared with (1*S*,2*S*)-EMPS as the SDA] and 13 crystals of (−)-GTM-3 [prepared with (1*R*,2*R*)-EMPS as the SDA] (Table 1). One of the typical reconstructed 3DED data of GTM-3 is shown in Figure S10 in the Supporting Information. Each data set was processed separately using PETS2.0 software,^37^ including peak search, determination of the orientation matrix, optimization of the frame orientation, and determination of integrated reflection intensities. After procession, the output file of.cif_pets was used for structure solution and kinematical refinement, and the output file of _dyn.cif_pets was used for dynamical refinement using the new version of Jana 2020.^38^ More details of the data procession and dynamical refinement can be found in the tutorials of PETS2 and Jana 2020 softwares.^39^ After performing dynamical refinement on the individual 15 data sets (Table S2 in the Supporting Information), 13 crystals of (+)-GTM-3 [prepared with (1*S*,2*S*)-EMPS] were identified as polymorph *P*4_3_32 as significantly lower R-factors were found, while the 2 remaining crystals were identified as polymorph *P*4_1_32. For the 13 crystals of (−)-GTM-3 [prepared with (1*R*,2*R*)-EMPS as SDA] (Table S3 in the Supporting Information), 10 of them were identified as polymorph *P*4_1_32, and the remaining 3 were identified with polymorph *P*4_3_32. Please note that the SDAs in the pores of GTM-3 were not considered during the dynamical refinement on the framework structure of GTM-3 because of the light scattering of the organic SDA compared with the inorganic frameworks. The unit cell parameters were modified by Powley fitting against SPXRD data during dynamical refinement. These results indicate that GTM-3 zeolite samples obtained using EMPS as SDA are not enantio-pure but instead are enantio-enriched to an average ratio of 82%. Interestingly, the absolute configuration coincides with that predicted by our computational catalytic model, providing confidence in the models used for the reaction mechanism study.

**Table 1:** 3D-ED Determination of the Absolute Configuration of GTM-3 Crystals

sample	SDA	number of crystals			enrichment *P*4_1_32/*P*4_3_32
		analyzed	*P*4_1_32	*P*4_3_32	
(+)-GTM-3	(1*S*,2*S*)-EMPS	15	2	13	13/87
(−)-GTM-3	(1*R*,2*R*)-EMPS	13	10	3	77/23

**Figure 8:**
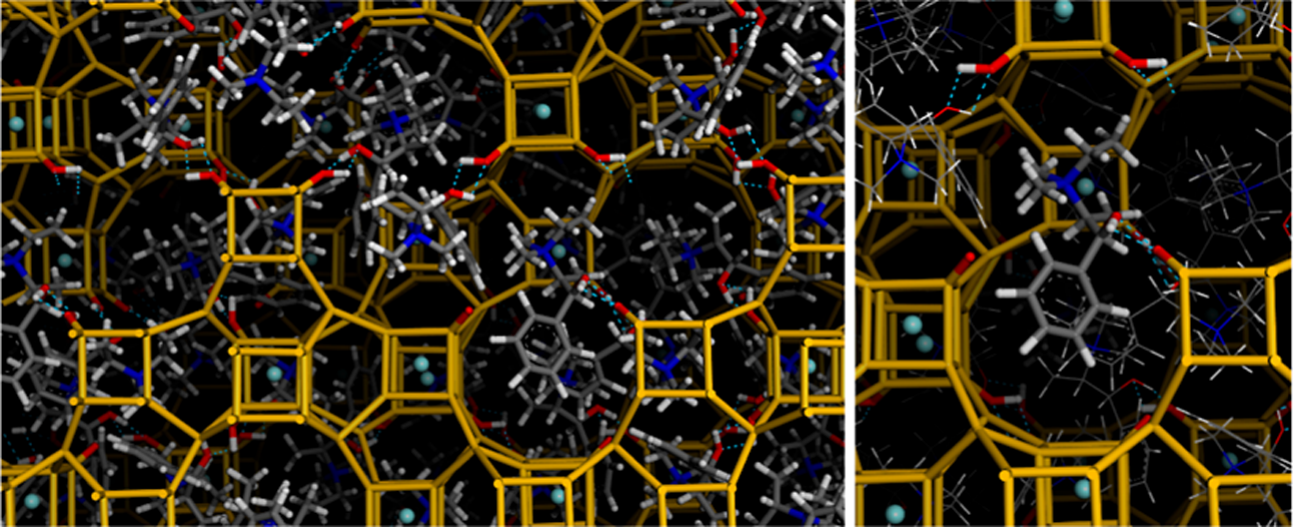
Two views of the location of *SS*-EMPS within the *P*4_3_32 -ITV polymorph, highlighting the transfer of chirality occurring through the formation of H-bonds with Ge(7)OH groups of *d4r*-A units; framework O atoms are omitted for clarity; H-bonds are displayed as dashed blue lines.

We finally combined the simulated annealing routine with Rietveld refinement to determine the location of the guest species, both F and, importantly, *SS*-EMPS cations, within the *P*4_3_32 -ITV framework; Figure S11 and Table S4 in the Supporting Information provide details of the structure-refinement. Figure 8 shows the locations of the *SS*-EMPS cations in the *P*4_3_32 framework (24 cations per unit cell). First no clustering (no π···π type interactions) between the aromatic ring of adjacent molecules was found, in agreement with the lack of dimer bands in the UV–vis fluorescence spectra.^17^ Interestingly, we found O(EMPS)···O(Ge(7)OH) distances of 2.586 Å, indicating the formation of strong host−guest H−bonds between hydroxyl groups of the organic chiral cations and of Ge(T7)OH groups in *d4r*-A units (see Figure 8-right); in contrast, no H-bond interaction is observed for Ge(T8)OH. Indeed, a close size-match between the -ITV cavities and the EMPS dimensions can be noticed (Figure 8-top-right). A mirror-image case is found for *RR*-EMPS in the *P*4_1_32 space group, with O(EMPS)···O(Ge(7)OH) distances of 2.615 Å (Figure S12 in the Supporting Information). The conformation adopted by EMPS within the framework is different as the most stable one, which might explain a shift of the ^13^C NMR band corresponding to C5 (see Figure S13 in the Supporting Information). On the other hand, F sites in the center of the *d4r* units, as expected.

**Figure 9:**
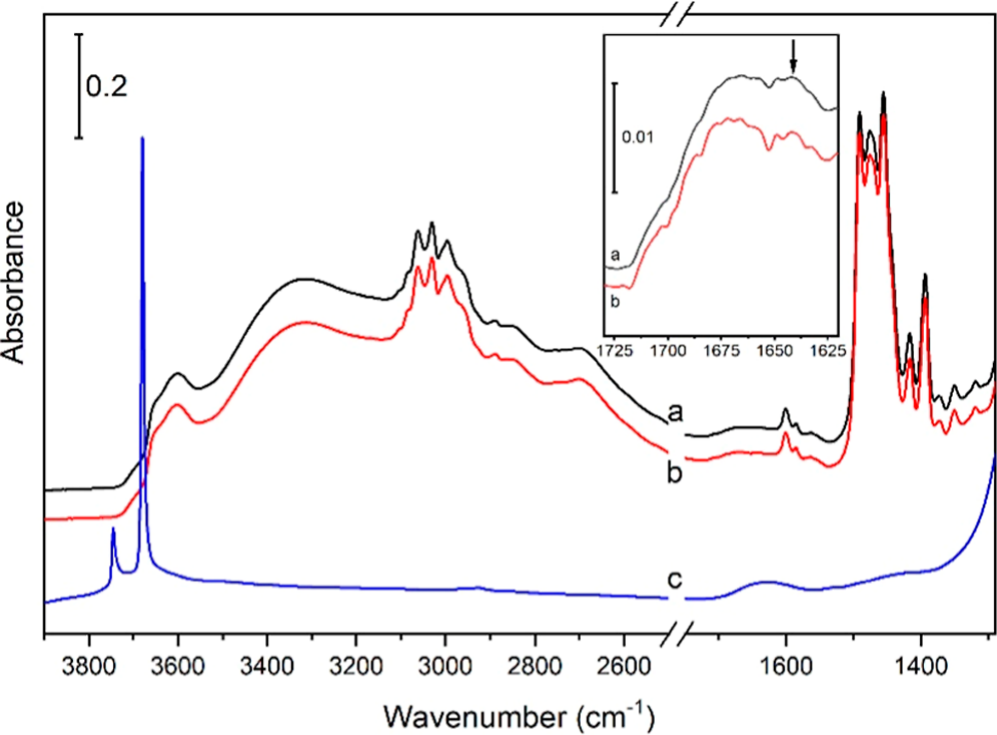
FTIR spectra of as-made GTM-3 degassed at room temperature for 5 min (a) and 1 h (b) and calcined GTM-3 degassed at 250 °C (c). Inset: enlarged region showing the virtual absence of the H–O–H bending band due to adsorbed water in the as-made sample (arrow) and its complete removal after 1 h degassing at room temperature.

Our results clearly show the crucial role of the development of host–guest H-bonds for the transfer of chirality from the organic chiral SDA to the -ITV framework to occur. In order to verify the formation of such H-bonds, we performed a FTIR study of sample (+)-GTM-3 (prepared with *SS*-EMPS) subjected to different treatments. FTIR spectrum of the calcined sample (without organics) shows two intense and sharp bands at 3745 and 3679 cm^–1^ (Figure 9-c) corresponding, respectively, to ≡Si–OH and ≡Ge–OH free groups. However, in the presence of the SDAs (as-made sample, spectra a and b in Figure 9), a broadening and red shift of these bands are observed, giving rise to a shoulder at *ca*. 3700 cm^–1^ (≡Si–OH) and two broad overlapping bands at ca. 3650 and 3600 cm^–1^ (assigned to ≡Ge–OH). On the other hand, the as-made sample seems to contain a negligible amount of adsorbed water as the spectrum recorded after a short degassing at room temperature only shows a very weak shoulder at 1640 cm^–1^ (indicated by an arrow in Figure 9, inset), which is fully removed after 1 h degassing. Therefore, it can be concluded that the observed shifting of hydroxyl bands in the as-made sample is not due to the interaction with adsorbed water. The fact that, in the presence of the SDA, the ≡Ge–OH band is red-shifted and split into two bands suggests the presence of two distinct ≡Ge–OH groups developing interactions with the SDA molecules with different strengths: one ≡Ge–OH displays a weak interaction (shifted from 3679 to 3650 cm^–1^), which should correspond to Ge(T8)OH groups interacting with the aromatic rings, and another more abundant ≡Ge–OH displaying a much stronger interaction (shifted from 3679 to 3600 cm^–1^), which is assigned to Ge(T7)OH H-bonded with *SS*-EMPS. Thus, this FTIR study confirms the role of host–guest H-bonds between chiral SDAs and ≡Ge–OH framework groups during the transfer of chirality, which takes place through Ge in the T7 position.

## 4 Conclusions

Our combined experimental and computational study about the asymmetric catalytic activity of GTM-3 chiral zeolite catalysts has enabled the understanding of the origin of the enantioselectivity associated with these novel catalytic materials in the reaction between racemic *trans*-stilbene oxide and 1-butanol. Results show a particular orientation of the TSO enantiomers upon adsorption on Ge sites associated with the T7 interrupted positions, orientation that is determined by the development of an intraframework H-bond triggered by the distortion of the Ge environment that took place to accommodate the additional ligand as well as by the interaction with nearby hydroxyl framework groups. Such a particular orientation of the adsorbed reactant determines the facility for the attack of 1-butanol to the oxirane ring through an S_N_2 mechanism and of the subsequent required rotation of the butanol moiety to enable the final H-transfer to give the final *unlike* products. Our computational model suggests that *P*4_1_32 polymorphs should favor the S_N_2 route for the *SS*-TSO reactant to give mainly the *SR*-*unlike* products, while such a reaction path is strongly hindered for *RR*-TSO, and hence, this proceeds alternatively through a S_N_1 mechanism to give *RR*-*like* and secondary products, which is indeed favored for this TSO enantiomer. Comparison with the experimental characterization of the different reactant and product chiral species obtained from both enantiomorphic GTM-3 catalysts revealed that the (1*R*,2*R*)-enantiomer of the organic used for the crystallization of these materials (*N*,*N*-ethyl-methyl-pseudoephedrinium) should favor the *P*4_1_32 polymorph [and vice versa; *P*4_3_32 polymorph should be favored for the antipode (1*S*,2*S*)-enantiomer], allowing for the prediction of the absolute configuration of GTM-3 catalysts. Indeed, the determination of the absolute configuration of 28 individual crystals by 3DED has enabled the experimental confirmation of such an assignment of the chirality of GTM-3, giving a chiral enantio-enrichment of 82%. Moreover, determination of the location of the *SS*-enantiomer of the SDA revealed the crucial role of the development of H-bonds between hydroxyl groups of the organic chiral SDAs and the hydroxyl groups associated with the interrupted Ge T7 positions for the transfer of chirality in the host–guest system.

## Supporting Information Available

Details of the experimental analytical procedures, additional catalytic and characterization results, additional pictures and computational energy profiles, and details of the dynamical refinements and of the Rietveld refinement (PDF)

## References

[ref1] Nandi N. Chirality in Biological Nanospaces, Reactions in Active Sites; CRC Press, 2012.

[ref2] Rossino G.; Robescu M. S.; Licastro E.; Tedesco C.; Martello I.; Maffei L.; Vincenti G.; Bavaro T.; Collina S. BiocatalysisBiocatalysis: A smart and green tool for the preparation of chiral drugs A smart and green tool for the preparation of chiral drugs. Chirality 2022, 34, 1403–1418. 10.1002/chir.23498. 35929567 PMC9805200

[ref3] Heitbaum M.; Glorius F.; Escher I. Asymmetric Heterogeneous Catalysis. Angew. Chem., Int. Ed. 2006, 45, 4732–4762. 10.1002/anie.200504212. 16802397

[ref4] Yu Murzin D.; Mäki-Arvela P.; Toukoniitty E.; Salmi T. Asymmetric Heterogeneous Catalysis: Science and Engineering. Catal. Rev. 2005, 47, 175–256. 10.1081/CR-200057461.

[ref5] Davis M. E. Reflections on Routes to Enantioselective Solid Catalysts. Top. Catal. 2003, 25, 3–7. 10.1023/B:TOCA.0000003093.74240.23.

[ref6] Yu J.; Xu R. Chiral zeolitic materials: structural insights and synthetic challenges. J. Mater. Chem. 2008, 18, 4021–4030. 10.1039/b804136a.

[ref7] Davis M. E. A Thirty-Year Journey to the Creation of the First Enantiomerically Enriched Molecular Sieve. ACS Catal. 2018, 8, 10082–10088. 10.1021/acscatal.8b03080.

[ref8] van Erp T.; Caremans T. P.; Dubbeldam D.; Martin-Calvo A.; Calero S.; Martens J. A. Enantioselective adsorption in achiral zeolites. Angew. Chem., Int. Ed. 2010, 49, 3010–3013. 10.1002/anie.200906083. 20301152

[ref9] Castillo J. M.; Vlugt T. J. H.; Dubbeldam D.; Hamad S.; Calero S. Performance of Chiral Zeolites for Enantiomeric Separation Revealed by Molecular Simulation. J. Phys. Chem. C 2010, 114, 22207–22213. 10.1021/jp1079394.

[ref10] Morris R. E.; Bu X. H. Induction of chiral porous solids containing only achiral building blocks. Nat. Chem. 2010, 2, 353–361. 10.1038/nchem.628. 20414234

[ref11] Dubbeldam D.; Calero S.; Vlugt T. J. H. Exploring new methods and materials for enantioselective separations and catalysis. Mol. Sim. 2014, 40, 585–598. 10.1080/08927022.2013.829225.

[ref12] Davis M. E.; Lobo R. F. Zeolite and molecular sieve synthesis. Chem. Mater. 1992, 4, 756–768. 10.1021/cm00022a005.

[ref13] Smit B.; Maesen T. L. M. Towards a molecular understanding of shape selectivity. Nature 2008, 451, 671–678. 10.1038/nature06552. 18256663

[ref14] Moliner M.; Boronat M. Towards “enzyme-like” zeolite designs to maximize the efficiency of catalysts by molecular recognition: Fine-tuning confinement and active site location. Microporous Mesoporous Mater. 2023, 358, 112354 10.1016/j.micromeso.2022.112354.

[ref15] Gómez-Hortigüela L.; Bernardo-Maestro B. Chiral Organic Structure-Directing Agents. In Insights into the Chemistry of Organic Structure-Directing Agents in the Synthesis of Zeolitic Materials. Structure and Bonding; Gómez-Hortigüela L., Ed.; Springer: Cham, 2017; Vol. 175; pp 201–244. 10.1007/430_2017_9.

[ref16] Brand S. K.; Schmidt J. E.; Deem M. W.; Daeyaert F.; Ma Y.; Terasaki O.; Orazov M.; Davis M. E. Enantiomerically enriched, polycrystalline molecular sieves. Proc. Natl. Acad. Sci. U.S.A. 2017, 114, 5101–5106. 10.1073/pnas.1704638114. 28461490 PMC5441830

[ref17] de la Serna R.; Nieto D.; Sainz R.; Bernardo-Maestro B.; Mayoral Á.; Márquez-Álvarez C.; Pérez-Pariente J.; Gómez-Hortigüela L. GTM-3, an extra-large pore enantioselective chiral zeolitic catalyst. J. Am. Chem. Soc. 2022, 144, 8249–8256. 10.1021/jacs.2c01874. 35502872 PMC9100664

[ref18] Sala A.; Jordá J. L.; Sastre G.; Llamas-Saiz A. L.; Rey F.; Valencia S. Sugar-based synthesis of an enantiomorphically pure zeolite. Nat. Commun. 2024, 15, 5298 10.1038/s41467-024-49659-2. 38906859 PMC11192950

[ref19] Kubota Y.; Helmkamp M. M.; Zones S. I.; Davis M. E. Properties of organic cations that lead to the structure-direction of high-silica molecular sieves. Microporous Mater. 1996, 6, 213–229. 10.1016/0927-6513(96)00002-8.

[ref20] Gómez-Hortigüela L.; Camblor M. A. Introduction to the Zeolite Structure-Directing Phenomenon by Organic Species: General Aspects. In Insights into the Chemistry of Organic Structure-Directing Agents in the Synthesis of Zeolitic Materials. Structure and Bonding; Gómez-Hortigüela L., Ed.; Springer: Cham, 2013; Vol. 175.

[ref21] Moliner M.; Rey F.; Corma A. Towards the rational design of efficient organic structure-directing agents for zeolite synthesis. Angew. Chem., Int. Ed. 2013, 52, 13880–13889. 10.1002/anie.201304713. 24115577

[ref22] Kang J. H.; McCusker L. B.; Deem M. W.; Baerlocher C.; Davis M. E. Further Investigations of Racemic and Chiral Molecular Sieves of the STW Topology. Chem. Mater. 2021, 33 (5), 1752–1759. 10.1021/acs.chemmater.0c04573.

[ref23] Gálvez P.; Bernardo-Maestro B.; Vos E.; Díaz I.; López-Arbeloa F.; Pérez-Pariente J.; Gómez-Hortigüela L. ICP-2: A New Hybrid Organo-Inorganic Ferrierite Precursor with Expanded Layers Stabilized by π–π Stacking Interactions. J. Phys. Chem. C 2017, 21, 24114–24127. 10.1021/acs.jpcc.7b08377.

[ref24] Bernardo-Maestro B.; Gálvez P.; González D.; López-Arbeloa F.; Pérez-Pariente J.; Gómez-Hortigüela L. Conformational Space of (1R,2S)-Dimethyl-Ephedrinium and (1S,2S)-Dimethyl-Pseudoephedrinium in the Synthesis of Nanoporous Aluminophosphates. J. Phys. Chem. C 2018, 122, 20377–20390. 10.1021/acs.jpcc.8b06098.

[ref25] Nieto D.; Pérez-Pariente J.; Toran E.; López-Arbeloa F.; Gómez-Hortigüela L. Conformational sieving effect of organic structure-directing agents during the synthesis of zeolitic materials. Microporous Mesoporous Mater. 2019, 287, 56–64. 10.1016/j.micromeso.2019.05.052.

[ref26] Sun J.; Bonneau C.; Cantín Á.; Corma A.; Díaz-Cabañas M. J.; Moliner M.; Zhang D.; Li M.; Zou X. The ITQ-37 mesoporous chiral zeolite. Nature 2009, 458, 1154–1157. 10.1038/nature07957. 19407798

[ref27] Gómez-Hortigüela L.; Pérez-Pariente J.; Nieto Hernández D.; Bernardo Maestro M. B.; de la Serna Valdés R.; Sainz Vaque R. Enantioenriched chiral microporous material, preparation method and uses. WO 2021/219914 A1, 2021.

[ref28] de la Serna R.; Pérez-Pariente J.; Gómez-Hortigüela L. Asymmetric catalysis within chiral zeolitic nanospaces: chiral host-guest match in GTM-3 zeolite. Catal. Today 2024, 426, 114389 10.1016/j.cattod.2023.114389.

[ref29] de la Serna R.; Arnaiz I.; Márquez-Álvarez C.; Pérez-Pariente J.; Gómez-Hortigüela L. Inversion of chirality in GTM-4 enantio-enriched zeolite driven by a minor change of the structure-directing agent. Chem. Commun. 2022, 58, 13083–13086. 10.1039/D2CC04958A. 36349553

[ref30] Gómez-Hortigüela L.; Pérez-Pariente J.; de la Serna Valdés R.; Arnaíz Canos I.; Jurado Sánchez J. GTM-4 Enantio-enriched chiral microporous material, preparation method and uses. WO 2024/023380 A1, 2024.

[ref31] de la Serna R.; Jurado-Sánchez J.; Márquez-Álvarez C.; Pérez-Pariente J.; Gómez-Hortigüela L. A chiral zeolite material with improved enantioselective catalytic properties prepared from readily accessible ephedrine alkaloids. Microporous Mesoporous Mater. 2024, 371, 113083 10.1016/j.micromeso.2024.113083.

[ref32] Perdew J. P.; Ruzsinszky A.; Csonka G. I.; Vydrov O. A.; Scuseria G. E.; Constantin L. A.; Zhou X.; Burke K. Restoring the Density-Gradient Expansion for Exchange in Solids and Surfaces. Phys. Rev. Lett. 2008, 100, 136406 10.1103/PhysRevLett.100.136406. 18517979

[ref33] Tkatchenko A.; Scheffler M. Accurate Molecular Van Der Waals Interactions from Ground-State Electron Density and Free-Atom Reference Data. Phys. Rev. Lett. 2009, 102, 073005 10.1103/PhysRevLett.102.073005. 19257665

[ref34] Fischer M.; Angel R. J. Accurate structures and energetics of neutral-framework zeotypes from dispersion-corrected DFT calculations. J. Chem. Phys. 2017, 146, 174111 10.1063/1.4981528. 28477591

[ref35] Gramatikov S. P.; Petkov P. St.; Vayssilov G. N. The relative stability of SCM-14 germanosilicate with different distributions of germanium ions in the absence and presence of structure-directing agents. Inorg. Chem. Front. 2022, 9, 3747–3757. 10.1039/D2QI00697A.

[ref36] Klar P. B.; Krysiak Y.; Xu H.; Steciuk G.; Cho J.; Zou X.; Palatinus L. Accurate structure models and absolute configuration determination using dynamical effects in continuous-rotation 3D electron diffraction data. Nat. Chem. 2023, 15, 848–855. 10.1038/s41557-023-01186-1. 37081207 PMC10239730

[ref37] Palatinus L.; Brázda P.; Jelínek M.; Hrdá J.; Steciuk G.; Klementová M. Specifics of the data processing of precession electron diffraction tomography data and their implementation in the program PETS2.0. Acta Crystallogr., Sect. B: Struct. Sci., Cryst. Eng. Mater. 2019, 75, 512–522. 10.1107/S2052520619007534. 32830709

[ref38] Petricek V.; Palatinus L.; Plasil J.; Dusek M. Jana2020 - a new version of the crystallographic computing system Jana. Z. Kristallogr. 2023, 238, 271–282. 10.1515/zkri-2023-0005.

[ref39] http://pets.fzu.cz/ (accessed September 17, 2024).

