# Supplementary material for: On the Origin of Enantioselectivity in Chiral Zeolite Asymmetric Catalyst GTM-3: Host–Guest Transfer of Chirality
Source: ACS Appl Mater Interfaces. 2024 Sep 25;16(40):54067–80. doi: 10.1021/acsami.4c14487 (PMC11472255; doi:10.1021/acsami.4c14487)
Supplement: Supplementary file 1 — am4c14487_si_001.pdf [file am4c14487_si_001.pdf]

Supporting Information

On the origin of enantioselectivity in chiral zeolite asymmetric catalyst GTM-3: host-guest transfer of chirality

*Ramón de la Serna,[a] Jaime Jurado-Sánchez,[a] Jian Li,[b] Carlos Márquez-Álvarez,[a] Joaquín Pérez-Pariente[a] and Luis Gómez-Hortigüela[a,]**

[a] Instituto de Catálisis y Petroleoquímica, Consejo Superior de Investigaciones Científicas (ICP-CSIC), c/ Marie Curie 2, 28049 Madrid, Spain. Email: lhortiguela@icp.csic.es

[b] State Key Laboratory of Coordination Chemistry, School of Chemistry and Chemical Engineering, Nanjing University, 210023, Nanjing, China

KEYWORD. Zeolite, chirality, enantioselective, asymmetric catalysis, absolute configuration.

Experimental Procedures

Synthesis of GTM-3 catalysts

Synthesis of (1*R*,2*R*)-(-)- and (1*S*,2*S*)-(+)-N,N-ethyl-methyl-pseudoephedrinium hydroxide (referred as (-)-EMPS and (+)-EMPS, respectively), used as structure-directing agents for the synthesis of GTM-3 materials, was described in our previous work,[1] starting from the corresponding commercially available chiral precursor, (1*R*,2*R*)-(-)-pseudoephedrine or (1*S*,2*S*)-(+)-pseudoephedrine (Sigma-Aldrich, 98%). Antipode GTM-3 zeolites were prepared following our previous recipe, from the corresponding (-)-EMPS or (+)-EMPS hydroxides.[1]

Asymmetric catalytic activity of GTM-3

After calcination at 500 °C under air for 10 hours, the *(-)-* and *(+)-*GTM-3 solids were used as acid-catalysts for the ring-opening of chiral *trans*-stilbene oxide with 1-butanol, which was reported in our previous work to show the highest enantioselectivity among a number of epoxides and alcohols of different size.[2] Catalytic experiments were performed with 20 wt % of catalyst (with respect to *trans*-stilbene oxide), and with 1 mg/ml of racemic *trans*-stilbene oxide solution in 1-butanol. The reaction was carried out at room temperature under agitation, and aliquots were extracted at different time intervals. All manipulation of calcined GTM-3 samples was carried out under inert (N_2) atmosphere (in a dry box) in order to avoid degradation of the framework. Evolution of the reaction to give the different products was monitored by HPLC with chiral stationary phase. Identification of the different enantiomers of the reactants and products (‘*unlike*’ and ‘*like*’) was accomplished by ring-opening of commercial enantiomerically-pure (2*R*,3*R*)-2,3-Diphenyloxirane ((*R*,*R*)-*trans*-stilbene oxide) (TRC) in 1-butanol homogeneously catalyzed with sulfuric acid, which gave (1*R*,2*S*)-2-butoxy-1,2-diphenyl-ethanol ‘*unlike*’ product (via S_N2) and (1*R*,2*R*)-2-butoxy-1,2-diphenyl-ethanol ‘*like*’ product (via S_N1).

Chiral products were analyzed by HPLC (Agilent 1260 Infinity II Series, with Quad Pump and DAD detector) using a chiral chromatographic column Chiralcel OD-H (Daicel, Chiralcel OD-H, 4.6mmØ, 250mmL, particle size of 5µm); purity of the peaks was analyzed by UV-VIS spectroscopy. The reaction products were analyzed using mobile phases of 94.5(heptane):5.5(isopropanol) (flow 1.2 ml/min) at 4 °C for *unlike* (*RS* and *SR*) and *like (RR* and *SS)* 2-butoxy-1,2-diphenyl-ethanol products (λ = 215 nm) and 95(heptane):5(isopropanol) (flow 1.2 ml/min) for reactants (*SS* and *RR trans*-stilbene oxide) (λ = 235 nm) and diphenylacetaldehyde (λ = 215 nm).

Computational study of the reaction mechanism

Due to the size and complexity of the catalytic system, a suitable large cluster consisting of 274 atoms was used as -ITV model (with 73 T atoms), with a composition of $Ge_3Si_{70}O_{106}(OH)_{15}(H_{t65})$, where interrupted Si positions were saturated with H atoms (H_t); the composition of the *d4r* unit where reaction takes place was $Ge_3Si_5O_{12}(OH)_2$. A preliminary set of calculations about the stability of Ge in the different positions of the *d4r* (there are two types of *d4r* units, one with two Q3 interrupted positions and another with only one) was performed in order to find the most appropriate configuration for the Ge atoms to analyze the reaction mechanism (in this case, using an appropriate cluster with 214 atoms, 62 T atoms). After the trends observed for the stability of Ge in the different positions, Ge atoms were located in both adjacent T7 positions, which

correspond to the interrupted Q3 positions (with a dangling OH group), and in an adjacent T6 position (see Figure S1 and S2) in the cluster $Ge_3Si_{70}O_{106}(OH)_{15}(H_{t65})$; this model is large enough as to simulate the chiral framework structure around the main *d4r* unit that contains the active sites where the reaction will take place. In order to preserve the -ITV framework structure during the calculations, only atoms in the Ge-containing *d4r* unit and up to its second coordination shell, as well as all the terminal OH groups, were allowed to relax during geometry optimizations, keeping fixed the rest of atoms. Calculations (geometry optimization and transition-state search calculations) were performed with the cluster model at the DFT+D level (PBEsol functional[3] and the Tkatchenko and Scheffler dispersion term;[4] this method has been shown to provide an excellent performance for modeling zeolite neutral systems).[5] We used a numerical basis set (DNP+, double numerical plus polarization with diffuse functions) with DMol3 code; this set of calculations allowed us to explore the vast and complex energy landscape of the reactions taking place within the catalyst. Calculations were performed with an -ITV cluster taken from the $P4_132$ polymorph. For the calculation of the transition states, an initial guess was estimated by selecting an appropriate interatomic distance as reaction coordinate (of the bond being formed), and performing geometry-optimizations at different fixed intervals of such interatomic distance. Structure and activation energies of the transition states were then calculated by using linear synchronous transit (LST) and quadratic synchronous transit (QST) methods, as implemented in DMol3. The structure of the transition state was confirmed by calculating vibrational frequencies to show the presence of a negative frequency corresponding to the atomic rearrangement taking place. Unless specified, energies are reported as relative internal energies (by subtracting the energy of the system with free (isolated) molecules); all energies are given in kcal/mol. In selected cases, estimation of the free energies was carried out by adding the entropic contribution at a given temperature (298 K) after calculation of the Hessian.

Electron Diffraction to determine absolute configuration of GTM-3

3DED data were collected on a FEI Tecnai F20 TEM equipped with a Detrics QuADRO detector (512×512 pixels, Pixel size:75 µm). A Fischione 2550 cryo transfer tomography holder (maximum tilt range: ±79°) was employed to load samples with liquid nitrogen, allowing for effective sample freezing to reduce the beam damage.

FTIR

Fourier transform infrared (FTIR) spectroscopy was carried out using a Thermo Nicolet Nexus 670 spectrometer provided with a MCT cryodetector. Spectra of self-supporting wafers were recorded in the 4000-650 cm^{-1} wavenumber range at 4 cm^{-1} resolution using a transmission cell made of quartz, provided with ZnSe windows and connected to a vacuum line. The as-made sample was pressed into a 13 mm diameter sample wafer (4 $mg\cdot cm^{-2}$ thickness), placed in the quartz cell and degassed under dynamic vacuum (residual pressure below 10^{-5} hPa) at room temperature for selected time periods before recording the spectra. Subsequently, the wafer was transferred to a glass reactor and calcined under dry air flow at 500 °C for 8 h. The wafer was then transferred to the FTIR cell in a glove box under dry nitrogen atmosphere preventing exposure of the sample to ambient air. The sample was then degassed in the cell under dynamic vacuum at 250°C for 1 h, cooled down and the FTIR spectrum recorded at 100°C.

Schemes

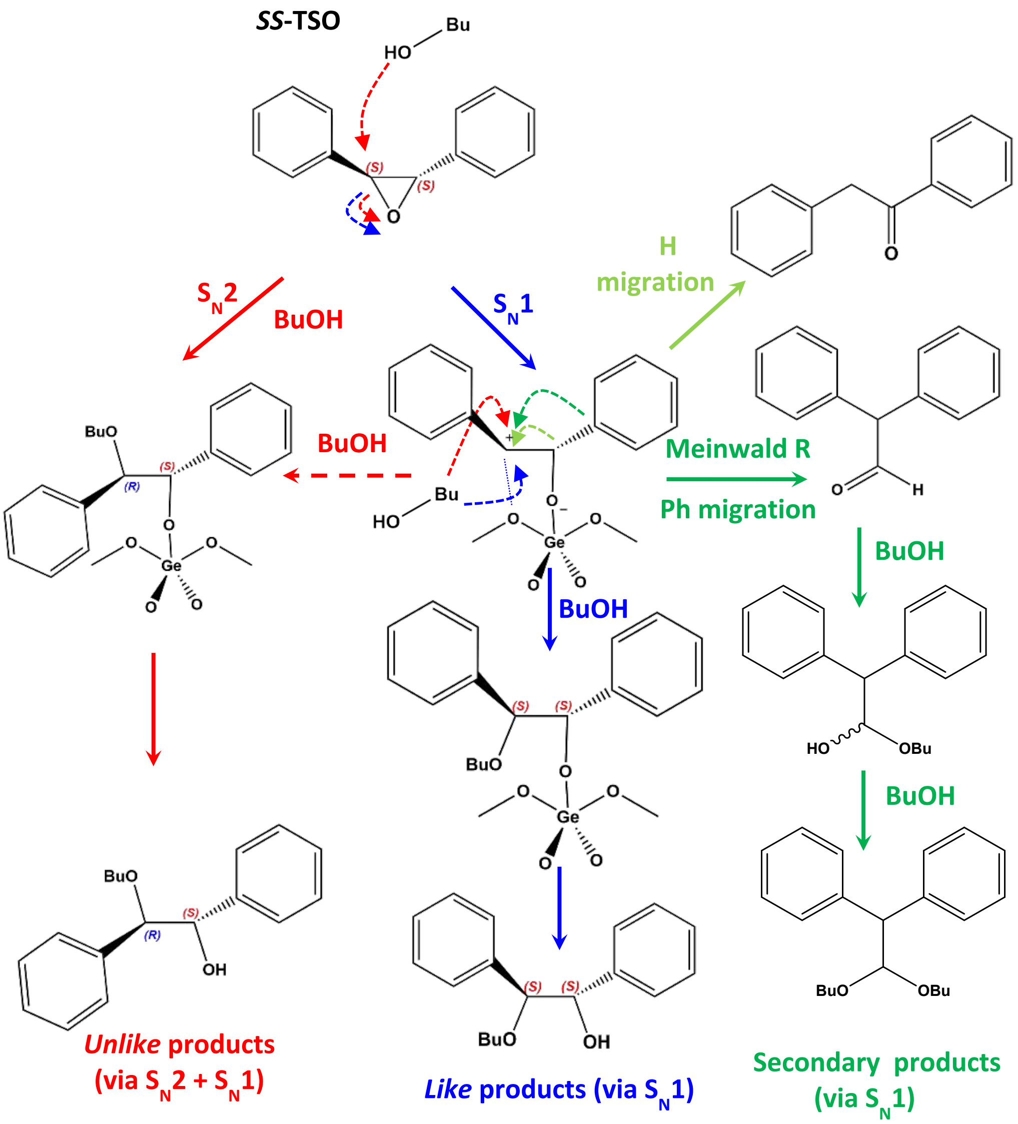

Scheme S1. Reaction mechanism proposed for the transformation of *SS*-TSO in the presence of GTM-3 catalysts via S_N2 or S_N1 routes.

Tables

Table S1. Internal relative energy (in kcal/mol Ge) for $Ge_1Si_{61}O_{104}(OH)_8Ht_{32}$ cluster models as a function of the position of Ge, located in *d4r*-A (T4, T5, T6 and T7), in *d4r*-B (T1, T2, T8 and T9) or in *lau* cages (no *d4r*) (T3, T10).

**Relative E (kcal/mol)**	***d4r*-A**	***d4r*-B**	**No *d4r/lau***
**[0-1]**	T7	T8	
**[1-2]**	T5, T6	T1	
**[2-3]**	T4	T2, T9	
**[3-6]**			T3, T10

Table S2. Details of the dynamical refinement of *(+)*-GTM-3 prepared from (1*S*,2*S*)-EMPS.

Microscope	FEI Tecnai F20									
Detector	Detrics QuADRO									
Radiation	electrons, 0.02508									
T(K)	99K									
Empirical formula	$Si_{192}O_{400}F_{20}$									
Z	1									
Crystal system	Cubic									
Unit cell dimensions	*a* = 26.2852(9) Å									
Crystal No.	1		2		3		4		5	
completeness	99.8		99.9		99.9		99.9		99.9	
Space group	P4(3)32	P4(1)32	P4(3)32	P4(1)32	P4(3)32	P4(1)32	P4(3)32	P4(1)32	P4(3)32	P4(1)32
Robs	0.174	0.273	0.205	0.283	0.192	0.301	0.182	0.277	0.195	0.296
Rall	0.250	0.365	0.261	0.392	0.291	0.384	0.257	0.398	0.301	0.381
wRall	0.339	0.392	0.351	0.421	0.368	0.431	0.323	0.413	0.352	0.414
Crystal No.	6		7		8		9		10	
completeness	99.9		99.8		99.8		99.8		99.9	
Space group	P4(3)32	P4(1)32	P4(3)32	P4(1)32	P4(3)32	P4(1)32	P4(3)32	P4(1)32	P4(3)32	P4(1)32
Robs	0.190	0.272	0.167	0.265	0.169	0.279	0.184	0.278	0.203	0.292
Rall	0.290	0.393	0.245	0.364	0.252	0.385	0.273	0.382	0.304	0.377
wRall	0.349	0.413	0.335	0.394	0.315	0.416	0.350	0.418	0.358	0.415
Crystal No.	11		12		13		14		15	
completeness	99.9		99.8		99.8		99.8		99.8	
Space group	P4(3)32	P4(1)32	P4(3)32	P4(1)32	P4(3)32	P4(1)32	P4(3)32	P4(1)32	P4(3)32	P4(1)32
Robs	0.200	0.287	0.180	0.275	0.284	0.187	0.267	0.168	0.189	0.263
Rall	0.254	0.386	0.269	0.391	0.375	0.296	0.375	0.248	0.289	0.367
wRall	0.356	0.436	0.326	0.437	0.412	0.333	0.392	0.321	0.331	0.394

Table S3. Details of the dynamical refinement of *(-)*-GTM-3 prepared from (1*R*,2*R*)-EMPS.

Microscope	FEI Tecnai F20									
Detector	Detrics QuADRO									
Radiation	electrons, 0.02508									
T(K)	99K									
Empirical formula	$Si_{192}O_{400}F_{20}$									
Z	1									
Crystal system	Cubic									
Unit cell dimensions	*a* = 26.2852(9) Å									
Crystal No.	1		2		3		4		5	
completeness	95.6		96.9		95.5		95.7		96.5	
Space group	P4(3)32	P4(1)32	P4(3)32	P4(1)32	P4(3)32	P4(1)32	P4(3)32	P4(1)32	P4(3)32	P4(1)32
Robs	0.281	0.213	0.261	0.201	0.301	0.217	0.319	0.202	0.266	0.189
Rall	0.354	0.271	0.395	0.291	0.356	0.279	0.358	0.264	0.362	0.298
wRall	0.423	0.329	0.411	0.315	0.416	0.332	0.447	0.337	0.428	0.328
Crystal No.	6		7		8		9		10	
completeness	97.9		97.6		97.5		96		97.8	
Space group	P4(3)32	P4(1)32	P4(3)32	P4(1)32	P4(3)32	P4(1)32	P4(3)32	P4(1)32	P4(3)32	P4(1)32
Robs	0.304	0.200	0.271	0.221	0.304	0.201	0.291	0.218	0.252	0.181
Rall	0.397	0.294	0.396	0.273	0.397	0.294	0.366	0.293	0.372	0.268
wRall	0.438	0.312	0.427	0.432	0.438	0.312	0.441	0.351	0.431	0.336
Crystal No.	11		12		13					
completeness	96.6		96.2		95.3					
Space group	P4(3)32	P4(1)32	P4(3)32	P4(1)32	P4(3)32	P4(1)32				
Robs	0.182	0.264	0.208	0.285	0.217	0.296				
Rall	0.274	0.378	0.261	0.351	0.289	0.375				
wRall	0.334	0.425	0.338	0.435	0.341	0.436				

Table S4. Details of the Rietveld refinement.

Identification code	(+)-GTM-3 prepared from (1*S*,2*S*)-EMPS
Empirical formula	$[Si_{141.75}Ge_{50.25}O_{400}H_{24}]|(C_{247.62}H_{419.05}N_{19.05}O_{19.05})F_{20}$
Wavelength	0.61928 Å
Radiation	Synchrotron Radiation
Crystal system	Cubic
Space group	$P4_332$
Unit cell dimensions	*a* = 26.2852(9) Å
Volume	18160.9(5) Å^3
Z	1
2ϑ range for data refinement	$2° < 2\vartheta < 35°$
Number of parameters	135
Number of reflections	1763
Number of data points	11168
Number of restrains	45 for Si-O and 77 for O-Si-O and Si-O-Si
Refinement method	Rietveld refinement

$R_p/R_{wp}/R_{exp}$/GoF	7.44%, 9.66%, 5.44%,1.89

Figures

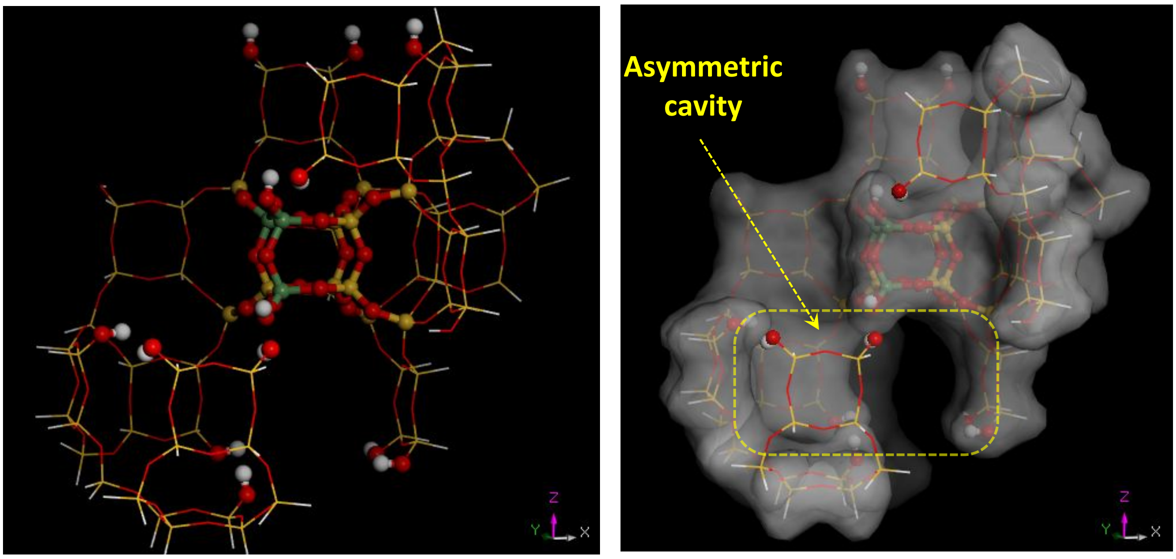

Figure S1. Cluster model of the -ITV structure used to study the reaction mechanism (P$4_1$32 enantiomer)

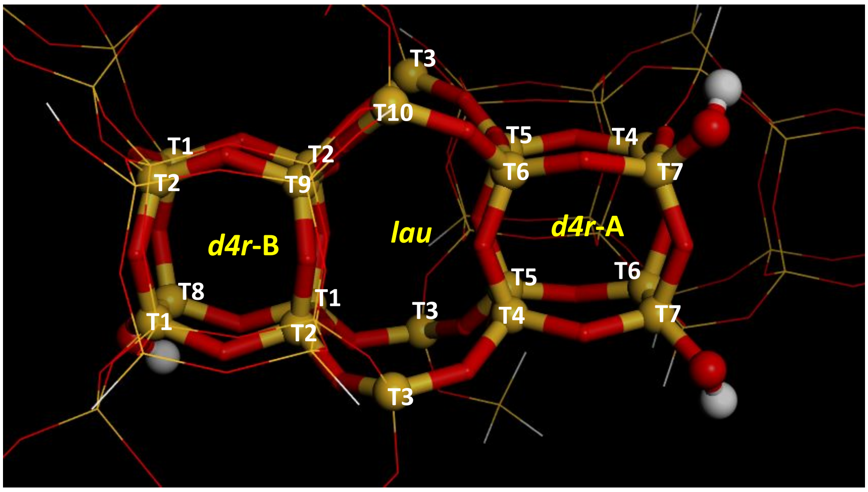

Figure S2. Crystallographic positions in the -ITV framework structure.

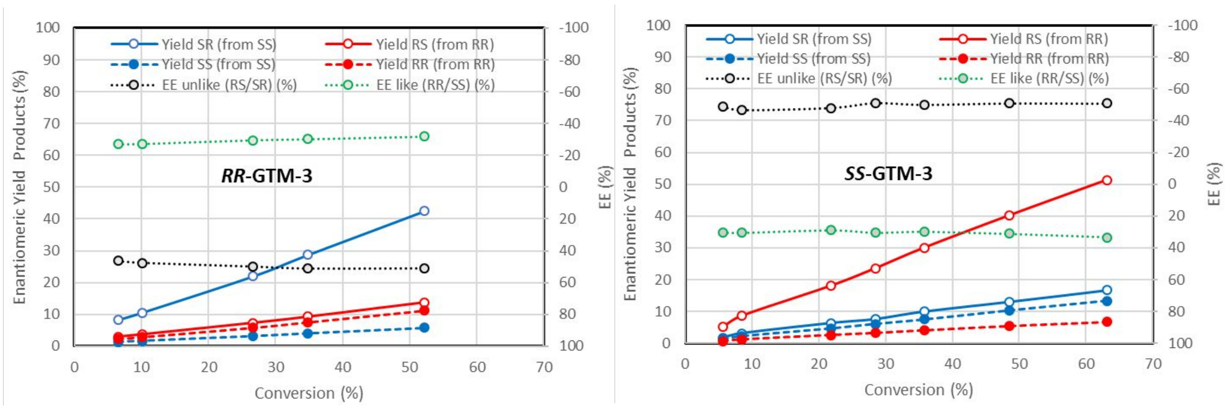

Figure S3. Yield of *unlike* (solid line) and *like* (dashed line) of *(-)*-GTM-3 (left) and *(+)*-GTM-3 (right) catalysts and corresponding enantiomeric excesses (black dotted line for *unlike* and green dotted line for *like* products); yields (blue and red lines) refer to the left axis, and *ee* values to the right axis.

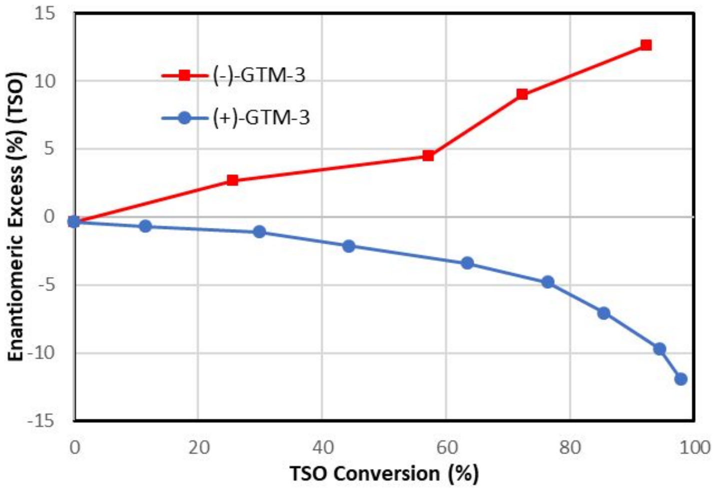

Figure S4. Enantiomeric excess of the remaining TSO reactant during the reaction in the absence of butanol (toluene as solvent) to give diphenylacetaldehyde.

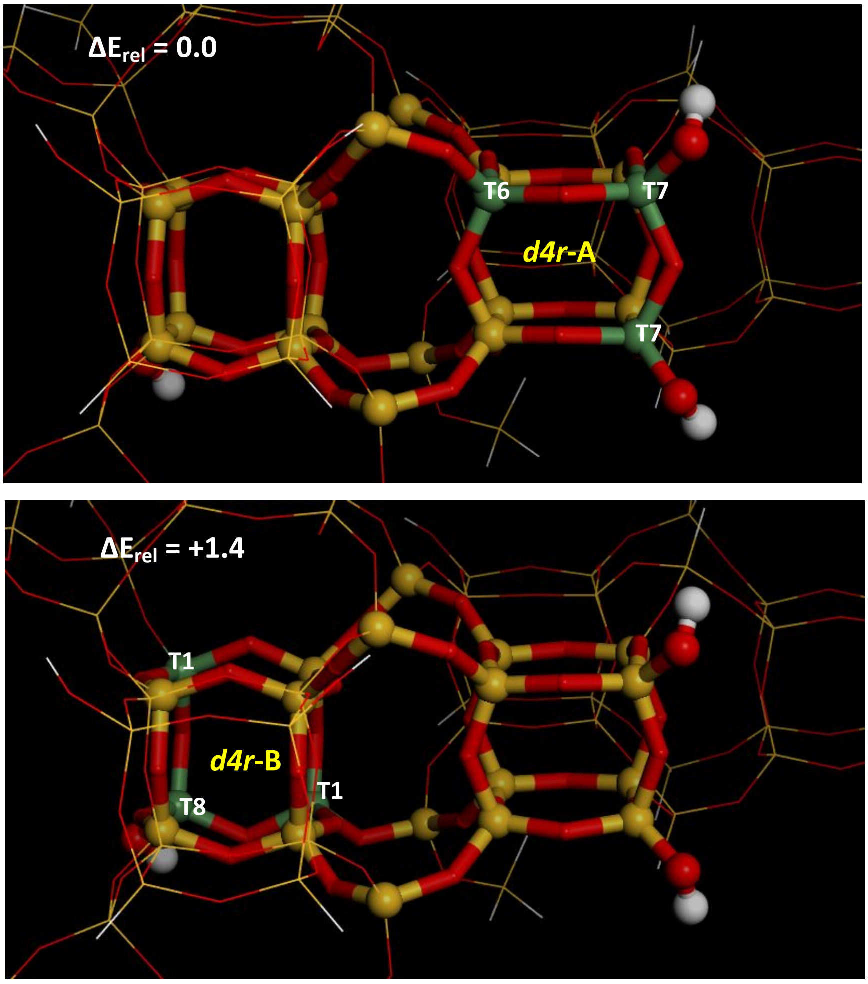

Figure S5. Most stable 3Ge-clusters in *d4r*-A (top) or *d4r*-B (bottom) units.

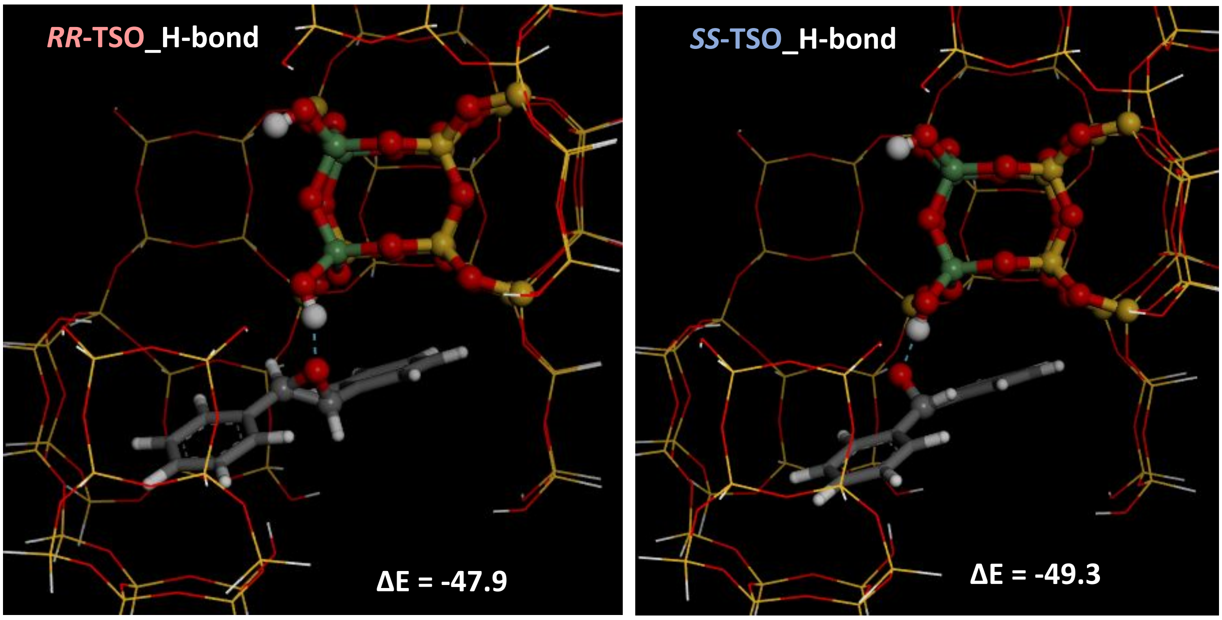

Figure S6. Geometry-optimized structure of *RR*-TSO (left) and *SS*-TSO (right) stabilized by H-bonds with framework OH groups.

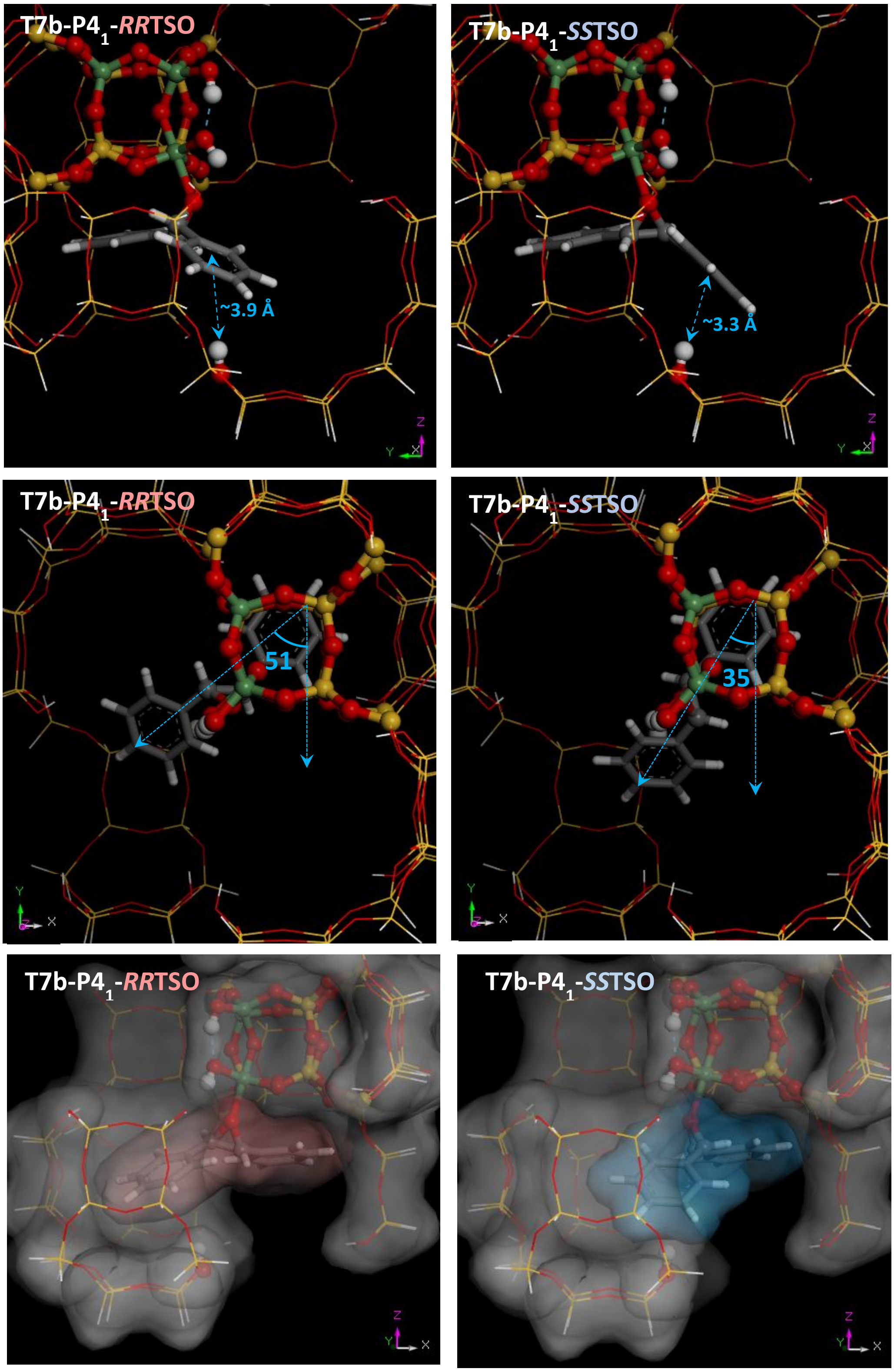

Figure S7. Three differently oriented views (top yz, middle xy, and bottom xz) of the Ge(T7)···TSO complexes with *RR*-TSO (left) and *SS*-TSO (right); Connolly surfaces are displayed at the bottom to highlight the host-guest match.

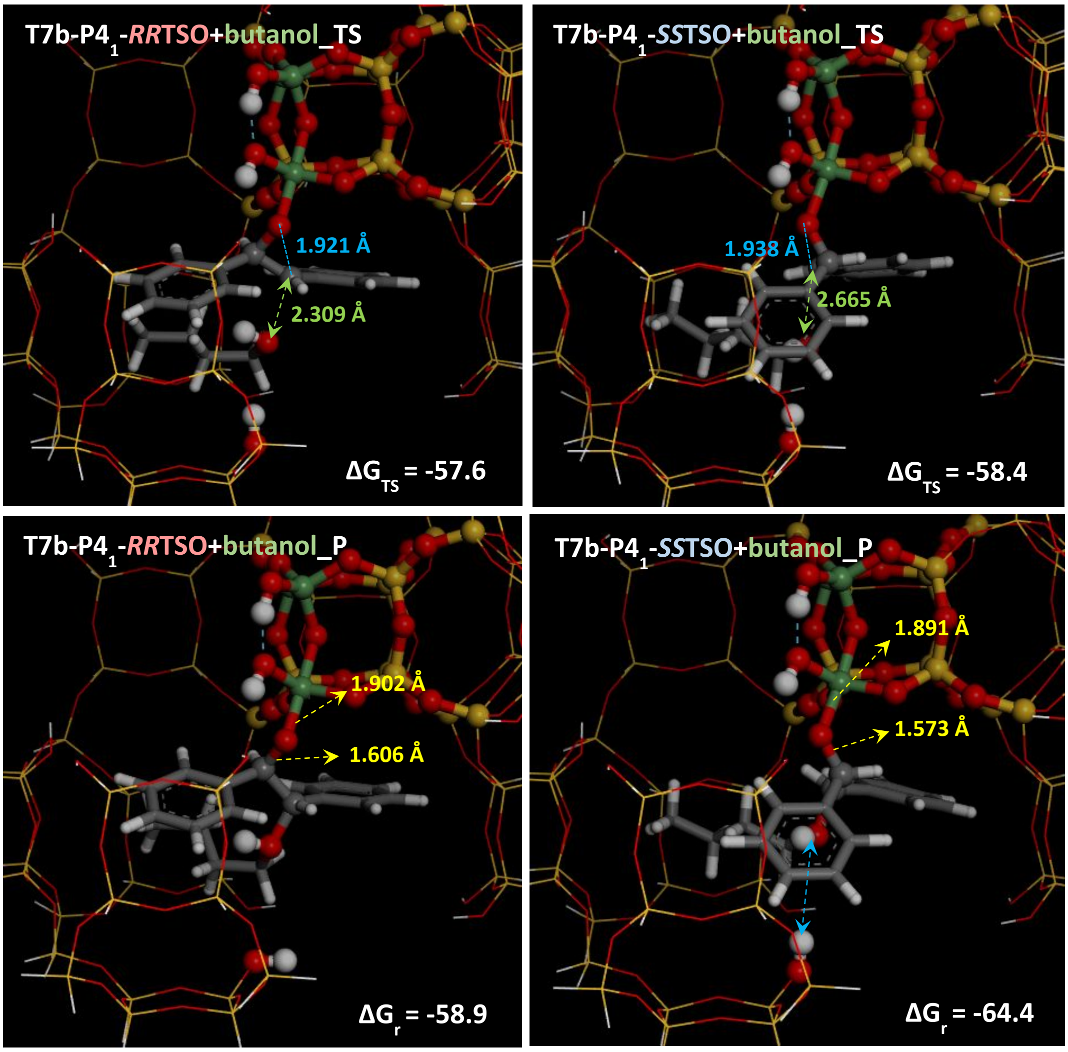

Figure S8. Bottom: geometry-optimized structure of the products after the S_N2 addition of butanol to the oxirane ring from *RR*- (left) or *SS*- (right) TSO.

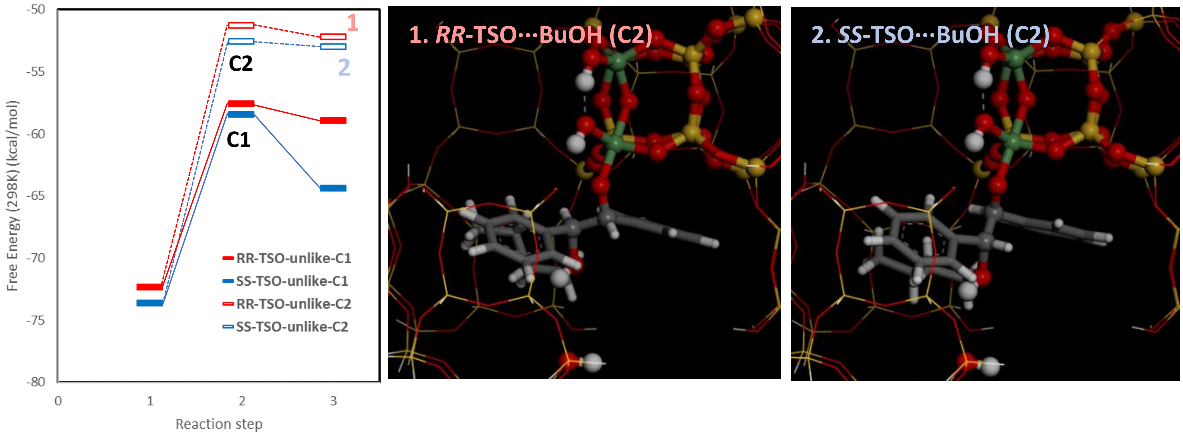

Figure S9. Comparison of the free energy profiles (left) for the addition of butanol to C1 (dashed lines) or to C2 (dotted lines) for *RR*-TSO (red lines) and *SS*-TSO (blue lines), and the structure of the final products (middle and right).

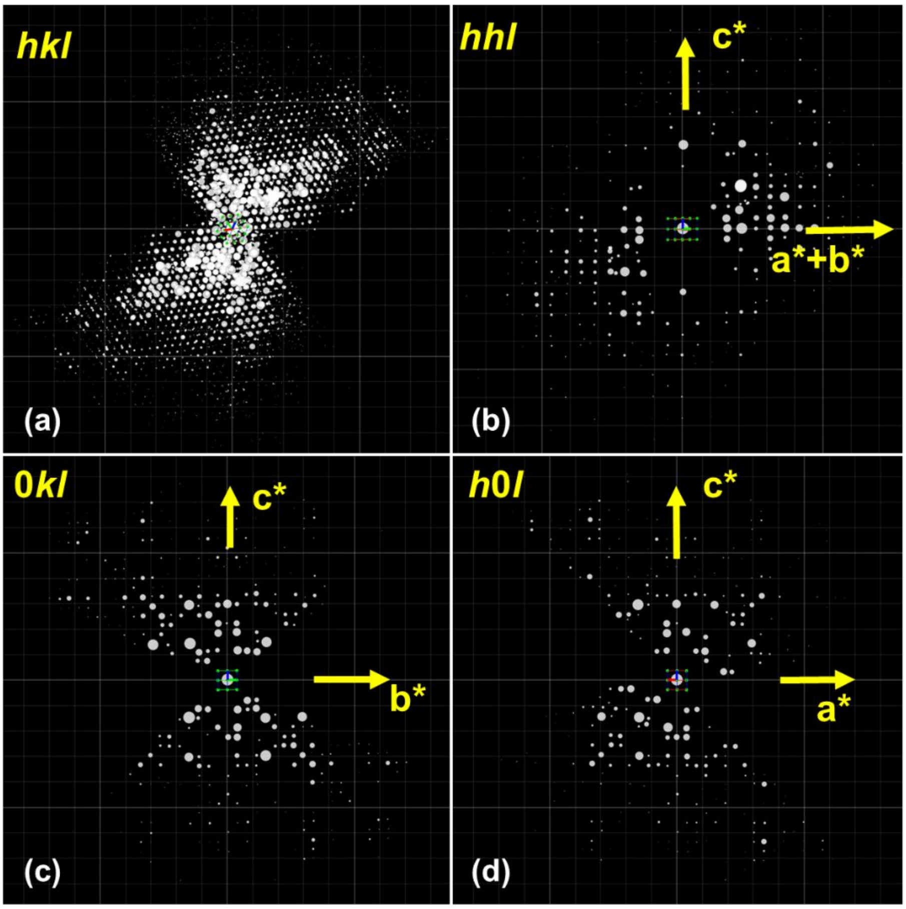

Figure S10. Typical reconstructed 3DED data of GTM-3 zeolite: (a) overview, and selected planes in the reciprocal lattice corresponding to (b) *hhl*, (c) 0*kl*, (d) *h*0*l*.

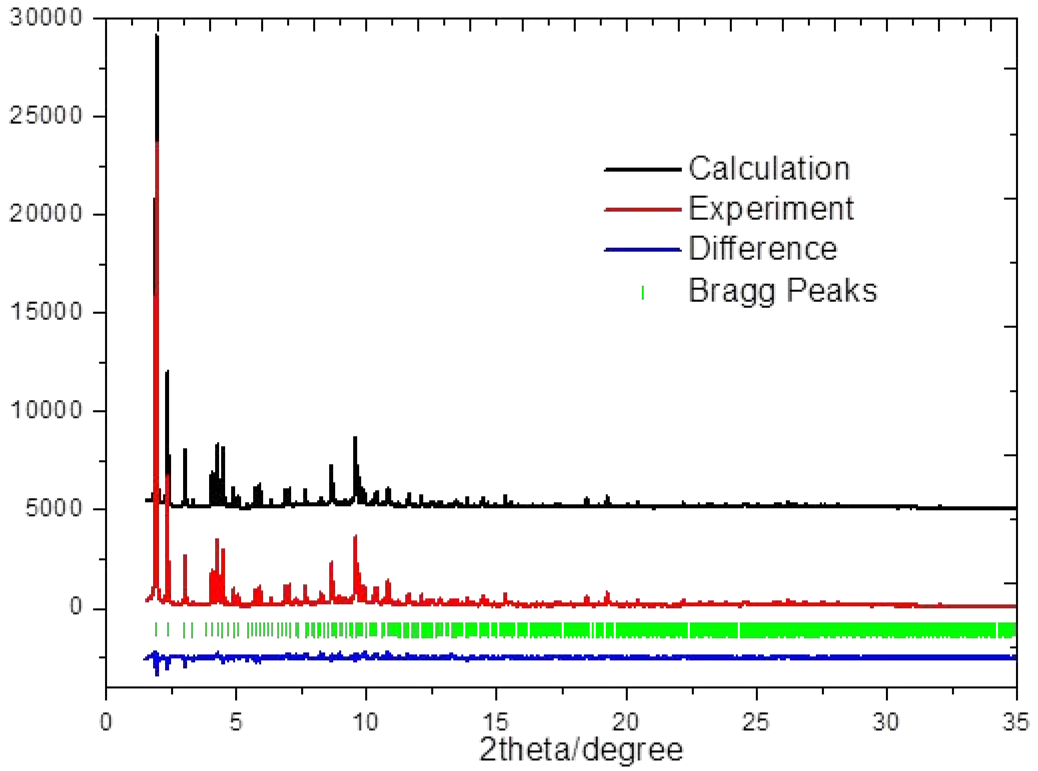

Figure S11. Rietveld refinement plots of *SS*-EMPS cations within the $P4_332$ -ITV framework with profile, weighted profile, and expected profile fitting factors R_p=7.44%, R_{wp}=9.66% and R_{exp}=5.44% respectively. The wavelength was λ = 0.61928.

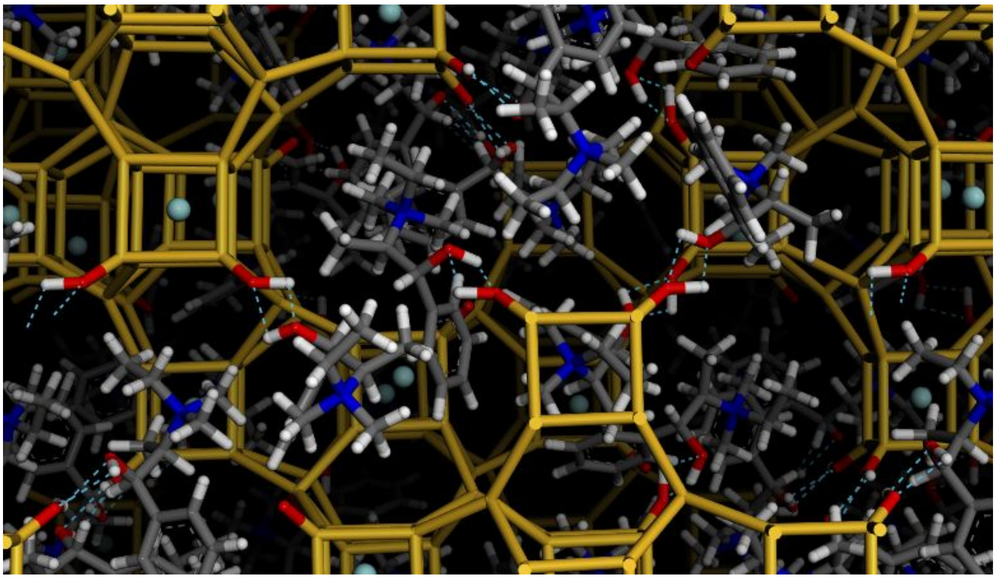

Figure S12. Location of *RR*-EMPS within the $P4_132$ -ITV polymorph; framework O atoms are omitted for clarity; H-bonds are displayed as dashed blue lines.

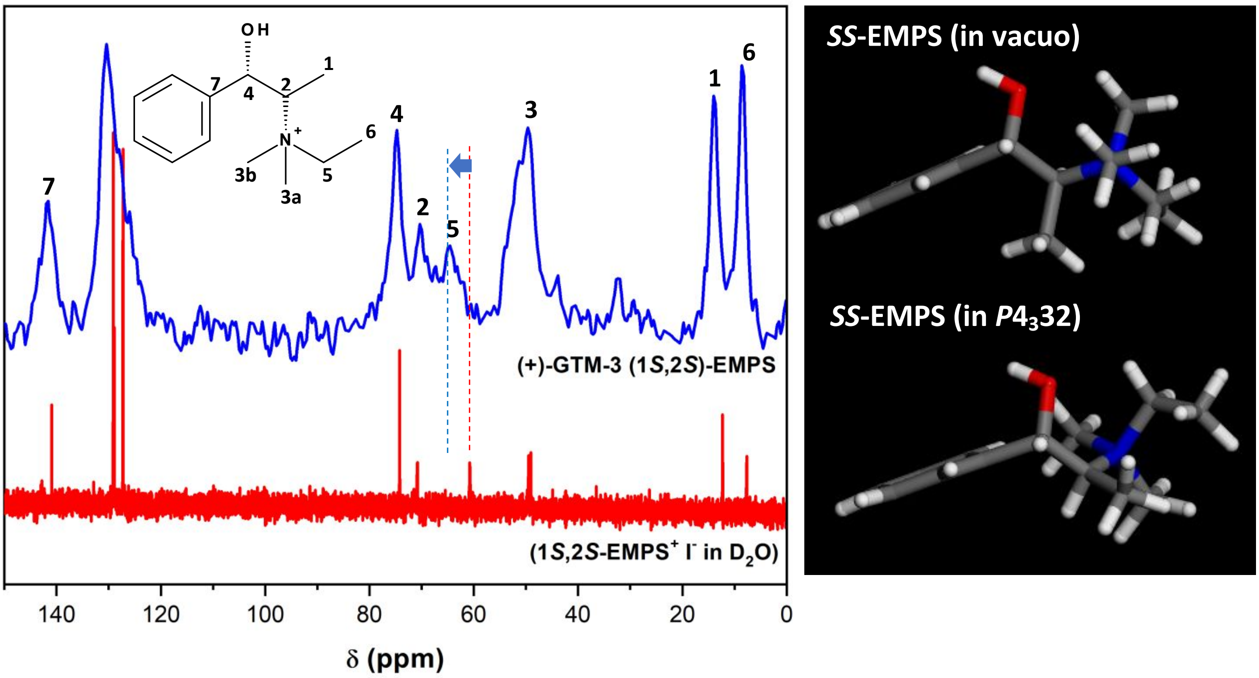

Figure S13. Left: ^{13}C CP MAS NMR of *(+)*-GTM-3 (prepared with (1*S*,2*S*)-EMPS) (top, blue line) and of (1*S*,2*S*)-EMPS iodide in D_2O solution (bottom, red line). Right: most stable conformation in vacuo (top) and conformation found within $P4_332$ -ITV (bottom).

References

(1) de la Serna, R.; Nieto, D.; Sainz, R.; Bernardo-Maestro, B.; Mayoral, Á.; Márquez-Álvarez, C.; Pérez-Pariente, J.; Gómez-Hortigüela, L. GTM-3, an extra-large pore enantioselective chiral zeolitic catalyst. *J. Am. Chem. Soc.* **2022**, *144*, 8249-8256.

(2) de la Serna, R.; Pérez-Pariente, J.; Gómez-Hortigüela, L. Asymmetric catalysis within chiral zeolitic nanospaces: chiral host-guest match in GTM-3 zeolite. *Catal. Today* **2024**, *426*, 114389.

(3) Perdew, J. P.; Ruzsinszky, A.; Csonka, G. I.; Vydrov, O. A.; Scuseria, G. E.; Constantin, L. A.; Zhou, X.; Burke, K. Restoring the Density-Gradient Expansion for Exchange in Solids and Surfaces. *Phys. Rev. Lett.* **2008**, *100*, 136406.

(4) Tkatchenko, A.; Scheffler, M. Accurate Molecular Van Der Waals Interactions from Ground-State Electron Density and Free-Atom Reference Data. *Phys. Rev. Lett.* **2009**, *102*, 073005.

(5) Fischer, M.; Angel, R. J. Accurate structures and energetics of neutral-framework zeotypes from dispersion-corrected DFT calculations. *J. Chem. Phys.* **2017**, *146*, 174111.
